# Lactylation: a novel epigenetic bridge connecting metabolic reprogramming and immune dysregulation in sepsis-associated ARDS

**DOI:** 10.3389/fimmu.2026.1879961

**Published:** 2026-07-01

**Authors:** Linxiao Wang, Lin Liu, Yanjun Wang, Fang Wang, Xi Gao, Yinghui Li

**Affiliations:** 1 Department of Clinical Laboratory, Honghui Hospital, Xi’an Jiaotong University, Xi’an, Shaanxi, China; 2 Departmentof Emergency, Honghui Hospital, Xi’an Jiaotong University, Xi’an, Shaanxi, China; 3 Department of Intensive Care Medicine, Honghui Hospital, Xi’an Jiaotong University, Xi’an, Shaanxi, China

**Keywords:** acute respiratory distress syndrome, epigenetics, histone lactylation, immune dysregulation, lactylation, metabolic reprogramming, post translational modification, sepsis

## Abstract

Sepsis−associated acute respiratory distress syndrome (ARDS) is driven by metabolic reprogramming and immune dysregulation, but the molecular link between them remains unclear. Lactylation, a lactate−derived post−translational modification, couples metabolic state to transcriptional and functional outcomes in immune and parenchymal cells as an epigenetic reader of glycolytic flux. Recent evidence demonstrates that lactylation regulates macrophage polarization, neutrophil extracellular trap formation, myeloid derived suppressor cell function, and T cell differentiation, while also controlling ferroptosis, autophagy, and endothelial injury in the septic lung. Clinical studies have identified histone H3K18 lactylation as a potential biomarker for sepsis severity and prognosis. This review establishes lactylation as a novel epigenetic bridge connecting metabolic reprogramming and immune dysregulation in sepsis associated ARDS and highlights therapeutic opportunities targeting this modification.

## 1 Introduction

Sepsis-associated acute respiratory distress syndrome (ARDS) remains a leading cause of mortality in critically ill patients, characterized by an uncontrolled inflammatory response and subsequent disruption of alveolar-capillary barrier integrity (1, 2). Specific therapies for sepsis−induced ARDS are still lacking, largely due to the complex interplay between metabolic dysregulation and immune dysfunction (3, 4). Metabolic reprogramming is a central hallmark of sepsis, where activated immune cells, especially macrophages, switch from oxidative phosphorylation to aerobic glycolysis, similar to the Warburg effect in cancer (5, 6). This glycolytic shift not only meets the bioenergetic demands of hyperactivated inflammatory cells but also leads to substantial lactate accumulation in the local microenvironment (7, 8). Once considered a waste product, lactate is now recognized as a signaling molecule and epigenetic modulator that bridges metabolism and gene expression (9).

Lactylation was first discovered in cancer research, where it was shown to function as an epigenetic reader of glycolytic flux. Zhang et al. (10) identified histone lysine lactylation as a modification that uses lactate−derived lactyl−CoA to deposit lactyl groups on histones, thereby promoting gene transcription. Subsequent cancer studies have established that lactylation regulates tumor cell proliferation, metastasis, immune evasion, and therapy resistance by remodeling both histone and non−histone proteins (11–13). A review have systematically elucidated how lactylation dynamically regulates the hallmarks of cancer, including sustained proliferative signaling, induction of angiogenesis, invasion and metastasis, and shaping of a proinflammatory tumor microenvironment (14). These investigations have also revealed that lactylation contributes to proliferation, therapy resistance, and immune evasion by influencing the function of regulatory T cells, macrophages, dendritic cells, and natural killer cells within the tumor microenvironment (15). Importantly, elevated lactate levels and histone lactylation have been detected in septic shock patients, with H3K18 lactylation proposed as a potential biomarker for disease severity (13). These observations suggest a compelling link between sepsis-induced metabolic reprogramming, lactylation, and the ensuing immune dysregulation that drives ARDS.

However, while lactylation has been extensively reviewed in oncology (16–20), its specific functions in sepsis−associated ARDS are only beginning to emerge. Unlike the chronic setting of cancer, sepsis presents an acute and dynamic metabolic−immune challenge. Lactylation in sepsis has dual effects: it may perpetuate hyperinflammation or facilitate immune resolution and tissue repair, depending on context and timing (21, 22). For example, H3K18 lactylation promotes endothelial glycocalyx degradation and exacerbates lung injury (23), yet it can also induce autophagic gene expression to mitigate immunosuppression (22). Such contrasting findings underscore the need for a comprehensive synthesis of current knowledge. Moreover, critical questions remain regarding the specific writers, erasers, and readers of lactylation in septic lung injury, as well as its interplay with other metabolic epigenetic marks such as acetylation and succinylation (24, 25).

Therefore, this review aims to systematically synthesize and critically evaluate the rapidly evolving evidence on lactylation as an epigenetic bridge connecting metabolic reprogramming and immune dysregulation in sepsis−associated ARDS. The metabolic underpinnings of lactate production during sepsis are first summarized, followed by a detailed discussion of the molecular machinery regulating histone and non−histone lactylation. Subsequently, the functional consequences of lactylation on key immune cell populations (macrophages, neutrophils, T cells) and its impact on alveolar epithelial and endothelial cells are analyzed. Finally, the translational potential of targeting the lactylation pathway is discussed, along with current knowledge gaps and future research directions. By bridging these areas, this review provides a conceptual framework for understanding how a metabolic byproduct orchestrates epigenetic and immune programs, offering new insights into the pathogenesis and treatment of sepsis−induced ARDS. Although ARDS can result from various etiologies including pneumonia, aspiration, trauma, and sepsis, sepsis remains the most common cause and is associated with the highest mortality. Therefore, this review focuses on sepsis−associated ARDS, while also discussing relevant findings from other ARDS models where applicable.

## 2 The biochemical and enzymatic basis of lactylation

**Figure 1:**
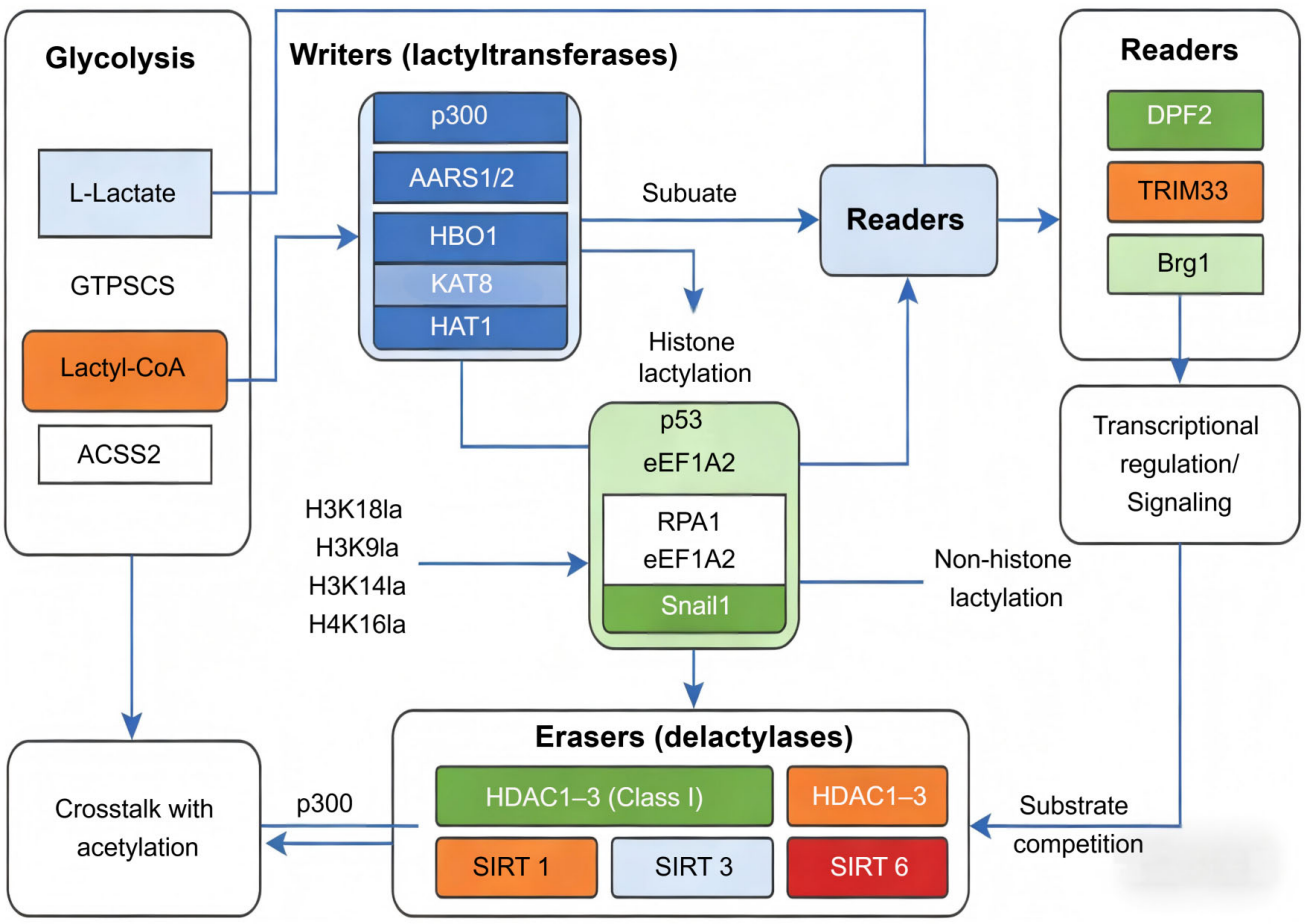
The biochemical and enzymatic basis of lactylation. Glycolysis-derived L-lactate is converted to lactyl-CoA by synthetases (GTPSCS, ACSS2). Writers (p300, AARS1/2, HBO1, KAT8, HAT1) transfer lactyl groups to histone and non-histone proteins. Erasers (HDAC1–3, SIRT1, SIRT3, SIRT6) remove lactyl groups. Readers (DPF2, TRIM33, Brg1) recognize lactyl-lysine to regulate transcription and signaling. Crosstalk with acetylation occurs via shared writers and erasers.

Lysine lactylation operates through a sophisticated enzymatic framework that transduces glycolytic flux into durable epigenetic and proteomic alterations. At the core of this regulatory system lies lactyl-CoA, the obligate acyl donor whose abundance directly reflects cellular lactate availability. A growing ensemble of writer enzymes catalyzes lactyl group deposition onto histones and non-histone substrates, while dedicated erasers ensure reversibility through nicotinamide adenine dinucleotide (NAD)-dependent or NAD-independent mechanisms. Emergent reader proteins interpret these marks to orchestrate transcriptional and signaling outcomes. Concurrently, extensive crosstalk with acetylation and other short-chain acyl modifications generates a combinatorial code that fine-tunes cellular responses to metabolic and inflammatory cues (**Figure 1**).

### 2.1 Chemical basis and substrate specificity

The biochemical foundation of histone lactylation rests upon the identification of lactyl-coenzyme A as the requisite acyl donor substrate. Seminal work by Zhang et al. (10) demonstrated that lactate-derived lactyl-CoA serves as the direct precursor for lysine lactylation, establishing a direct metabolic-epigenetic linkage wherein glycolytic flux governs substrate availability. A subsequent investigation by Zhang et al. (26) provided crucial stereochemical resolution, establishing that L-lactylation represents the predominant isomer induced under glycolytic conditions, whereas D-lactylation arises from distinct metabolic sources. This chiral specificity bears functional consequences, as the two enantiomers engage distinct subsets of writer and eraser enzymes. Furthermore, Moreno-Yruela et al. (27) elucidated that chiral posttranslational modifications to lysine ϵ-amino groups introduce an additional layer of regulatory complexity. The concentration of lactyl-CoA is intimately coupled to the cellular metabolic state, positioning lactylation as a rheostat that transduces fluctuations in glycolytic activity into durable epigenetic alterations. Recent work by Liu et al. (28) identified nuclear GTP−specific succinyl−CoA synthetase (GTPSCS) as a lactyl-CoA synthetase that promotes histone lactylation and gliomagenesis, revealing that lactyl-CoA production can be compartmentalized within the nucleus. Zhu et al. (29) similarly demonstrated that acyl-CoA synthetase short-chain family member 2 (ACSS2) functions as a lactyl-CoA synthetase, coupling with lysine acetyltransferase 2A (KAT2A) to facilitate histone lactylation and tumor immune evasion. These findings collectively underscore that substrate generation is not merely a passive consequence of cytosolic lactate accumulation but involves active enzymatic machinery that may be therapeutically tractable.

### 2.2 Writers: acyltransferases catalyzing lactylation

The enzymatic landscape responsible for lactyl moiety deposition has expanded considerably beyond initial characterizations. The histone acetyltransferase p300 was first implicated as a lactyltransferase by Zhang et al. (10). However, the writer repertoire is substantially broader. Li et al. (30) discovered that alanyl−tRNA synthetases alanyl-tRNA synthetase 1 (AARS1) and alanyl-tRNA synthetase 2 (AARS2) function as global lysine lactyltransferases, sensing L−lactate and lactylating diverse substrates. This finding was independently corroborated by Zong et al. (31), who demonstrated that AARS1 lactylates p53 and contributes to tumorigenesis. Zong et al. (32) further reviewed the emerging roles of lysine lactyltransferases, emphasizing the functional diversity of this enzyme class. Additional writers include histone acetyltransferase binding to ORC1 (HBO1), which Niu et al. (33) identified as a catalyst for histone H3K9 lactylation that regulates gene transcription. Xie et al. (34) reported that lysine acetyltransferase 8 (KAT8) catalyzes lactylation of eukaryotic translation elongation factor 1 alpha 2 (eEF1A2) to promote protein synthesis in colorectal carcinogenesis. The multiplicity of lactyltransferases raises important questions regarding functional redundancy versus specialization. While p300 operates broadly across histone substrates, AARS1/2 exhibit pronounced non-histone targeting, and HBO1 displays selectivity for specific histone residues. A recent investigation by He et al. (35) identified histone acetyltransferase 1 (HAT1) as a lactyltransferase that mediates replication protein A1 (RPA1) lactylation to promote DNA repair and radioresistance, further expanding the writer catalog. This division of labor may permit fine-tuned regulation wherein distinct metabolic inputs engage specific writer enzymes to achieve context-appropriate lactylation patterns.

### 2.3 Erasers: deacylases mediating delactylation

The dynamic nature of lactylation is ensured by eraser enzymes that catalyze the removal of lactyl groups. Moreno-Yruela et al. (36) established that Class I histone deacetylases histone deacetylase 1, 2, and 3 (HDAC1, HDAC2, HDAC3) function as robust histone lysine delactylases. Du et al. (37) identified sirtuin 1 (SIRT1) and SIRT3 as robust lysine delactylases, demonstrating that SIRT1-mediated delactylation regulates glycolytic flux. Fan et al. (38) specifically identified SIRT3 as an eraser of H4K16 lactylation. Chen et al. (39) provided further evidence that SIRT3 functions as an eraser of H3K9 lactylation to inhibit esophageal cancer progression. Nickel et al. (40) recently demonstrated that Sirtuin 6 is a histone delactylase, expanding the sirtuin family’s role in lactylation dynamics. The sirtuin family’s NAD dependence links lactylation erasure to cellular energetic status, suggesting that conditions of NAD depletion may impair delactylase function and thereby reinforce lactylation accumulation. Du et al. (37) further reported that SIRT1 and SIRT3 exhibit robust delactylase activity across multiple substrates. A critical unresolved question concerns the relative selectivity of these erasers for lactyl versus acetyl groups *in vivo*, as the extent to which cellular compartmentalization or post-translational regulation confers differential activity remains largely unexplored. Notably, Tsusaka et al. (41) confirmed that Class I HDACs catalyze lysine lactylation removal, reinforcing the dual functionality of these enzymes.

### 2.4 Readers: recognition domains interpreting lactylation

The functional translation of lactylation marks into biological outcomes depends on reader proteins that specifically recognize and bind lactylated lysine residues. Zhai et al. (42) identified the bromodomain-containing protein double PHD fingers 2 (DPF2) as a histone lactylation reader that drives transcription and tumorigenesis. Nuñez et al. (43) demonstrated that the tripartite motif−containing 33 (TRIM33) bromodomain recognizes histone lysine lactylation with specificity distinct from its acetyl-lysine recognition. Hu et al. (44) implicated Brg1 as a histone lactylation reader during early iPSC reprogramming. The identification of reader proteins remains in its relative infancy compared with the characterization of writers and erasers. Current evidence suggests that lactyl-lysine recognition may employ both canonical bromodomains and potentially dedicated recognition modules yet to be discovered. The ability of bromodomain proteins to discriminate between lactyl- and acetyl-lysine is likely conferred by subtle differences in binding pocket geometry. Systematic proteomic approaches are urgently needed to comprehensively map the lactylation interactome.

### 2.5 Crosstalk with acetylation and other acyl modifications

Lactylation engages in extensive crosstalk with other lysine acyl modifications, particularly acetylation. Zhao et al. (45) demonstrated that lactate modulates zygotic genome activation through H3K18 lactylation rather than H3K27 acetylation. Di et al. (46) proposed that the H3K18 lactylation-to-acetylation ratio may serve as a biomarker for sepsis severity. The shared utilization of p300 as a writer for both modifications, and of HDAC1–3 as erasers, establishes a biochemical basis for reciprocal regulation. Sheng et al. (25) provided a comprehensive review of histone L-lactylation biochemistry and regulation, emphasizing the integrated nature of the cellular acyl modification landscape. Crosstalk also extends to other acyl modifications including succinylation and crotonylation, which similarly derive from metabolic intermediates. A critical challenge is developing analytical methods capable of simultaneously quantifying multiple acyl modifications at single-lysine resolution, as bulk measurements obscure the combinatorial complexity underlying functional specificity. Recent work by Zhang et al. (26) characterizing the dominant role of L-lactylation under glycolytic conditions provides a foundation for dissecting these competitive relationships.

**Figure 2:**
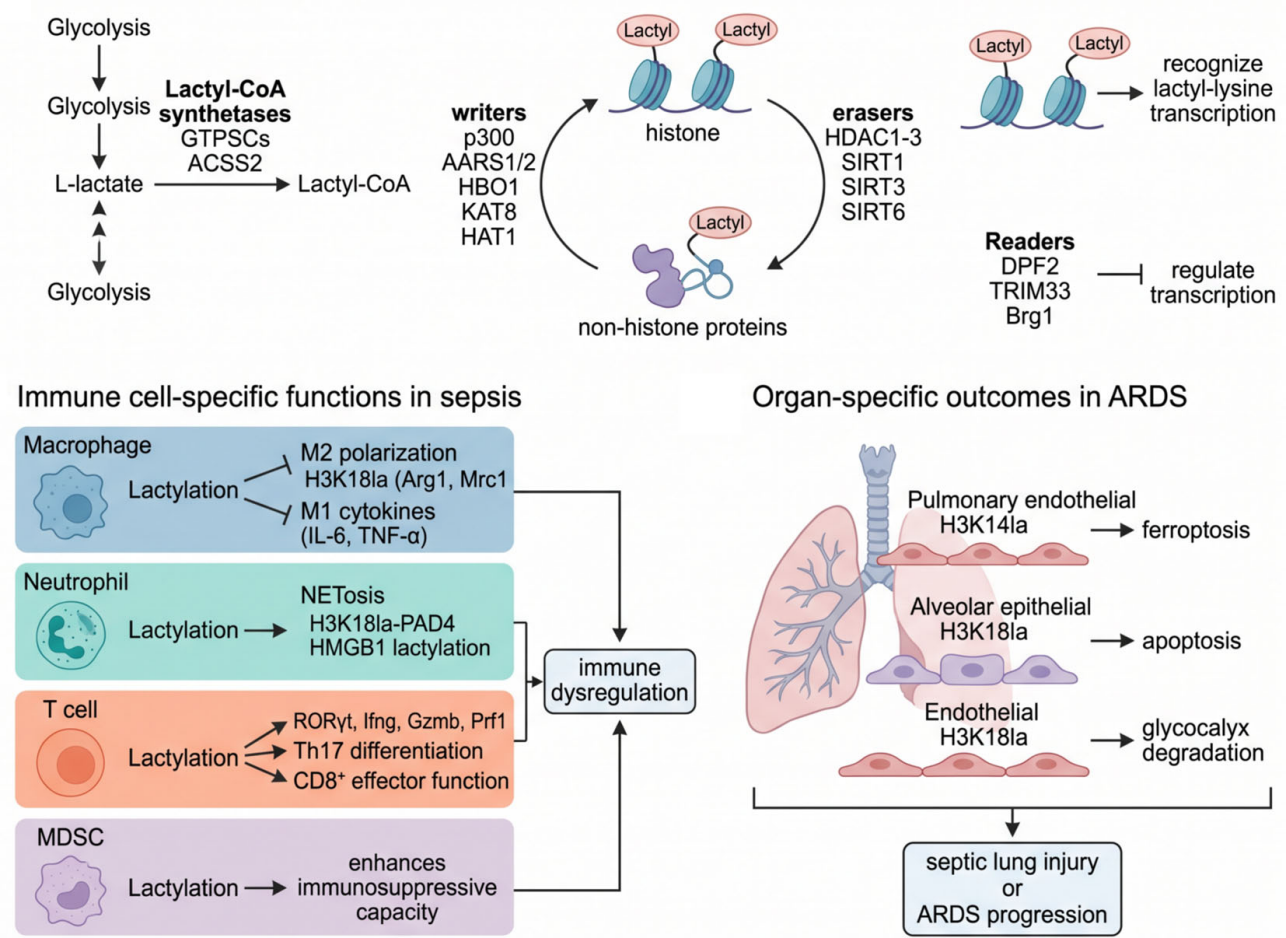
Schematic overview of lactylation machinery and its functional roles in immune cells during sepsis−associated ARDS. Glycolysis−derived L−lactate is converted to lactyl−CoA by synthetases (GTPSCS, ACSS2). Writers (p300, AARS1/2, HBO1, KAT8, HAT1) transfer lactyl groups to histone and non−histone proteins. Erasers (HDAC1−3, SIRT1, SIRT3, SIRT6) remove lactyl groups. Readers (DPF2, TRIM33, Brg1) recognize lactyl−lysine to regulate transcription. In macrophages, lactylation promotes M2 polarization (H3K18la at Arg1, Mrc1) and suppresses M1 cytokines. In neutrophils, lactylation drives NETosis via H3K18la−PAD4 and HMGB1 lactylation. In T cells, lactylation supports Th17 differentiation and CD8+ effector function. In MDSCs, lactylation enhances immunosuppressive capacity. Organ−specific outcomes in ARDS include pulmonary endothelial H3K14la−mediated ferroptosis, alveolar epithelial H3K18la−induced apoptosis, and endothelial H3K18la−driven glycocalyx degradation.

Mechanistically, lactylation and acetylation compete for the same lysine residues on histone tails. p300 exhibits higher catalytic efficiency for acetyl-CoA than for lactyl-CoA, meaning lactylation predominates only when lactate levels are substantially elevated, such as during sepsis (25). Conversely, Class I HDACs remove both acetyl and lactyl groups with comparable efficiency (36). This competition directly influences immune gene expression: H3K18 lactylation at pro−inflammatory gene promoters (e.g., IL−6, TNF−α) can replace acetylation, altering transcription factor recruitment and modulating inflammatory responses (46). Crosstalk also extends to succinylation, which is derived from succinyl−CoA and tends to inhibit metabolic enzyme activity, whereas lactylation is more prominent in glycolytically active immune cells (25). The balance among these modifications creates a “metabolic−epigenetic code” that fine−tunes NF−κB target gene expression and immune cell function during sepsis (47, 48). The enzymatic machinery of lactylation (writers, erasers, readers) and its cell−type−specific functions in immune cells during sepsis−associated ARDS are summarized schematically in **Figure 2**.

## 3 Lactylation in metabolic reprogramming and cellular processes

**Figure 3:**
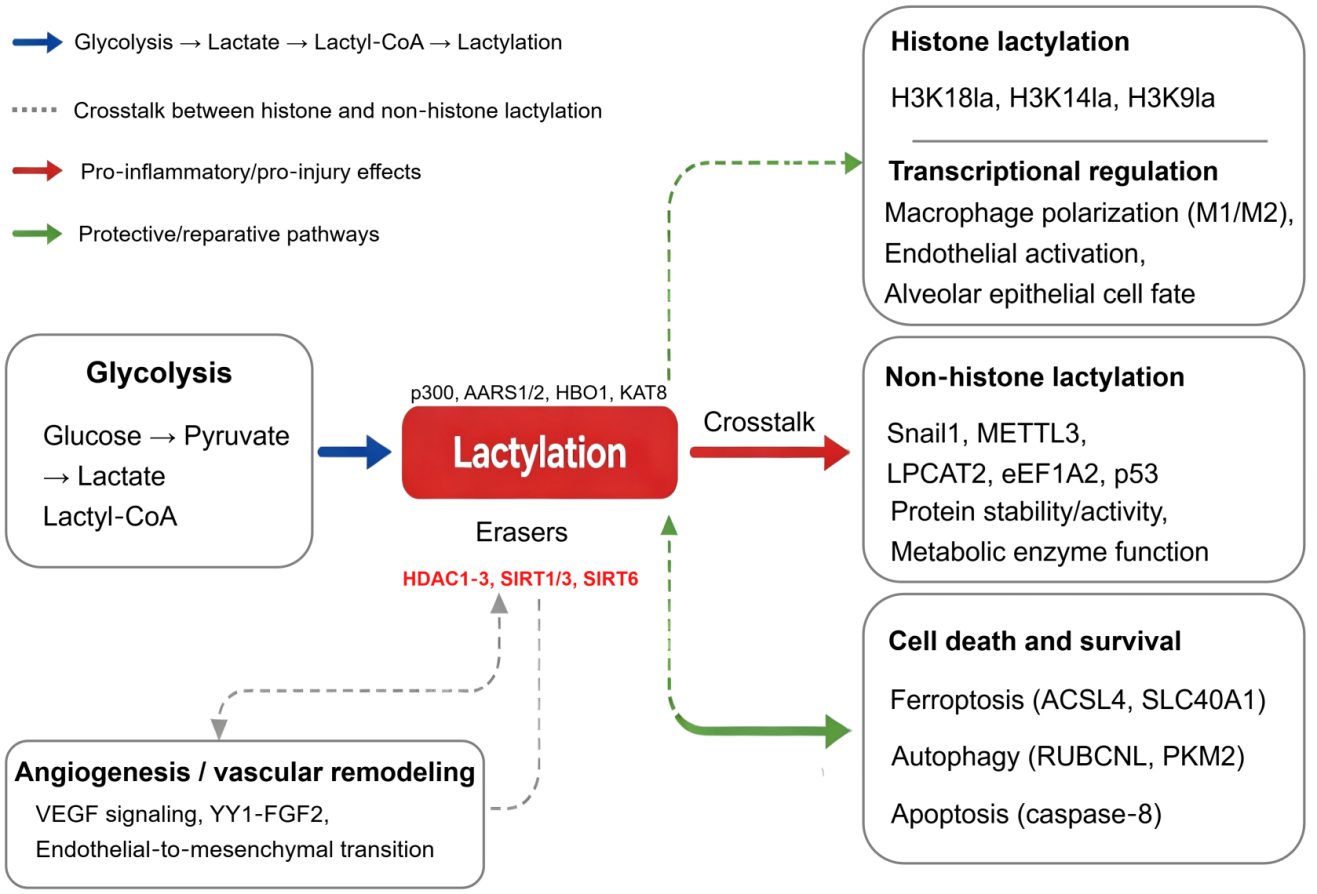
Lactylation couple glycolytic flux to transcriptional regulation, cell death, angiogenesis, and non-histone signaling in sepsis-associated ARDS. Lactylation acts as a metabolic−epigenetic sensor. Glycolysis−derived lactate drives histone lactylation (H3K18la, H3K14la, H3K9la) and non−histone lactylation (e.g., Snail1, METTL3, LPCAT2), regulating ferroptosis, autophagy, angiogenesis, and endothelial/macrophage function in the septic lung.

Lactylation functions as a pivotal sensor that directly couples glycolytic flux to transcriptional and functional outcomes across diverse cellular contexts. Accumulating evidence demonstrates that this modification serves not merely as a passive reflection of lactate abundance but as an active regulator of metabolic reprogramming, gene transcription, cell fate determination, and vascular adaptation. Histone lactylation at specific residues orchestrates context-dependent transcriptional programs, while non-histone lactylation modulates the activity of metabolic enzymes and signaling proteins. Furthermore, lactylation governs multiple cell death modalities and angiogenic processes, establishing a mechanistic framework for understanding its broader pathophysiological roles (**Figure 3**).

### 3.1 Lactylation as a sensor of glycolytic flux

The discovery of histone lactylation established a direct molecular conduit linking metabolic state to epigenetic regulation. Zhang et al. (10) demonstrated that lactate-derived lactyl-CoA serves as the direct substrate for lysine lactylation, positioning this modification as a rheostat that transduces fluctuations in glycolytic activity into durable chromatin alterations. This metabolic-epigenetic linkage is further reinforced by the observation that L-lactylation represents the predominant isomer induced under glycolytic conditions (49). Izzo and Wellen (50) emphasized that histone lactylation enables cells to sense changes in glucose metabolism and influence gene expression programs. Subsequent investigations have elucidated the mechanisms through which specific metabolic enzymes interface with the lactylation machinery. Rho et al. (51) demonstrated that hexokinase 2 mediated gene expression via histone lactylation is required for hepatic stellate cell activation and liver fibrosis, revealing a pathway wherein the initiating step of glycolysis directly influences fibrogenic transcriptional programs. Similarly, Li et al. (52) established a positive feedback loop between glycolysis and histone lactylation that drives oncogenesis in pancreatic ductal adenocarcinoma, illustrating how metabolic reprogramming and epigenetic remodeling reciprocally reinforce one another to sustain malignant phenotypes. Collectively, these findings underscore that lactylation functions as an integrated metabolic sensor rather than an isolated biochemical event. However, the precise threshold at which glycolytic flux triggers meaningful lactylation changes remains poorly defined, and whether distinct glycolytic intermediates beyond lactate contribute to modulating writer or eraser activity warrants systematic investigation.

### 3.2 Histone lactylation in transcriptional regulation

Histone lactylation at specific lysine residues exerts profound effects on transcriptional programs, with histone H3 lysine 18 lactylation (H3K18la), histone H3 lysine 14 lactylation (H3K14la), and histone H3 lysine 9 lactylation (H3K9la) emerging as particularly consequential marks. Yang et al. (53) demonstrated that hypoxic *in vitro* culture reduces histone lactylation and impairs preimplantation embryonic development in mice, providing early evidence that environmental conditions influencing lactate availability directly impact developmental gene expression through this modification. Subsequent work by Li et al. (54) revealed that hypoxia regulates fibrosis-related genes via histone lactylation in the placentas of patients with preeclampsia, linking oxygen tension, metabolic adaptation, and pathological gene activation. The transcriptional consequences of histone lactylation exhibit remarkable context specificity. In macrophages, Irizarry-Caro et al. (12) identified that the TLR signaling adapter BCAP regulates the inflammatory to reparatory transition by promoting histone lactylation, demonstrating that this modification facilitates phenotypic plasticity in innate immune cells. De Leo et al. (55) further showed that glucose-driven histone lactylation promotes the immunosuppressive activity of monocyte-derived macrophages in glioblastoma, revealing how the tumor microenvironment co-opts metabolic-epigenetic crosstalk to subvert immune surveillance. Contrasting these findings, Desgeorges et al. (56) reported that histone lactylation in macrophages is predictive for gene expression changes during ischemia-induced muscle regeneration, suggesting that the functional output of lactylation is highly dependent on cellular context and the repertoire of available transcription factors. A critical unresolved question concerns the mechanisms by which histone lactylation achieves gene-specific rather than global transcriptional effects. Current evidence suggests that sequence-specific transcription factors recruit lactyltransferases to discrete genomic loci, yet the full complement of such factors and their targeting logic remains incompletely characterized.

### 3.3 Non-histone lactylation

Beyond chromatin, lactylation of non-histone proteins has emerged as a critical regulatory mechanism that directly modulates the activity of metabolic enzymes and signaling molecules. Fan et al. (57) demonstrated that lactate promotes endothelial-to-mesenchymal transition via Snail1 lactylation after myocardial infarction, providing a paradigm wherein lactylation of a transcription factor alters cell state and tissue remodeling. This finding expanded the functional repertoire of lactylation beyond transcriptional regulation at histone substrates to include direct modulation of protein stability, localization, and activity. The scope of non-histone lactylation has since broadened considerably. Yu et al. (58) reported that lactylation of mitochondrial adenosine triphosphate synthase subunit alpha regulates vascular remodeling and progression of aortic dissection, revealing that this modification extends to organellar proteins and influences fundamental bioenergetic processes. Zhang et al. (59) comprehensively reviewed non-histone lactylation as a hub for tumor metabolic reprogramming and epigenetic regulation, cataloging diverse substrates and their functional consequences. Peng and Du (60) similarly provided a systematic overview of both histone and non-histone lactylation, emphasizing the molecular mechanisms and disease relevance of this modification. The functional diversity of non-histone lactylation raises important questions regarding substrate selectivity. Whereas histone lactylation appears largely governed by nuclear writer enzymes, non-histone lactylation may involve distinct compartmentalized machinery. Moreover, the extent to which non-histone lactylation occurs spontaneously versus through enzyme-catalyzed mechanisms remains debated, as the chemical reactivity of lactyl-CoA toward lysine residues under physiological conditions is not fully defined. Systematic proteomic approaches combined with genetic ablation of specific writers will be necessary to resolve these uncertainties.

### 3.4 Lactylation in cell death pathways

Lactylation exerts bidirectional control over multiple cell death modalities, including ferroptosis, autophagy, and apoptosis, with the net effect contingent upon cellular context and the specific proteins modified. Wu et al. (61) demonstrated that histone lactylation-regulated methyltransferase−like 3 (METTL3) promotes ferroptosis via m6A modification on ACSL4 in sepsis-associated lung injury, establishing a mechanistic link between metabolic reprogramming, epitranscriptomic regulation, and iron-dependent cell death. This pathway illustrates how lactylation can sensitize cells to ferroptotic stimuli under pathological conditions. Conversely, Li et al. (62) reported that tumor-derived lactate promotes resistance to bevacizumab treatment by facilitating autophagy enhancer protein rubicon like autophagy enhancer (RUBCNL) expression through H3K18 lactylation in colorectal cancer, revealing a pro-survival function wherein lactylation enhances autophagic flux to confer therapeutic resistance. Further expanding this paradigm, Xu et al. (63) showed that histone lactylation stimulated upregulation of proteasome 26S subunit, non−ATPase 14 (PSMD14) alleviates neuronal PANoptosis after traumatic brain injury, implicating lactylation in the integrated regulation of multiple cell death pathways. The contrasting roles of lactylation in promoting or suppressing cell death underscore the importance of context-dependent interpretation. In cancer cells, lactylation frequently supports survival and therapy resistance, whereas in inflammatory or ischemic settings, it may potentiate cell death programs. A critical challenge lies in determining whether these divergent outcomes reflect differences in the lactylation machinery itself or arise from cell-type-specific signaling networks that interpret lactylation marks. Longitudinal studies tracking lactylation dynamics during the progression from cell stress to death commitment will be essential for resolving these questions.

### 3.5 Lactylation in angiogenesis and vascular remodeling

Lactylation has emerged as a significant regulator of angiogenic processes and vascular homeostasis. Fan et al. (64) identified a feedback loop driven by H3K9 lactylation and HDAC2 in endothelial cells that regulates VEGF-induced angiogenesis, demonstrating that lactylation directly modulates the transcriptional response to pro-angiogenic stimuli. Wang et al. (65) further showed that Yin Yang 1 lactylation in microglia promotes angiogenesis through transcription activation-mediated upregulation of fibroblast growth factor 2 (FGF2), revealing that lactylation in non-endothelial cell types can indirectly orchestrate vascular growth through paracrine mechanisms. The clinical relevance of lactylation in vascular pathology is underscored by findings from Ma et al. (66), who reported that orphan nuclear receptor nuclear receptor subfamily 4 group A member 3 (NR4A3) promotes vascular calcification via histone lactylation, implicating this modification in pathological vascular remodeling. Zang et al. (67) demonstrated that isocitrate dehydrogenase 2 lactylation promotes angiogenesis in murine diabetic myocardial infarction, linking metabolic enzyme lactylation to compensatory neovascularization following ischemic injury. Collectively, these studies establish lactylation as a nodal point integrating metabolic cues with angiogenic transcriptional programs. However, several questions remain unresolved. The relative contributions of endothelial cell-intrinsic lactylation versus lactylation in supporting cells to overall angiogenic responses have not been systematically dissected. Furthermore, whether lactylation influences lymphangiogenesis through analogous mechanisms remains unexplored. Therapeutic targeting of lactylation in vascular disorders will require careful consideration of these context-dependent effects to avoid unintended consequences on physiological vascular homeostasis.

## 4 Lactylation in immune cell function and inflammation

**Figure 4:**
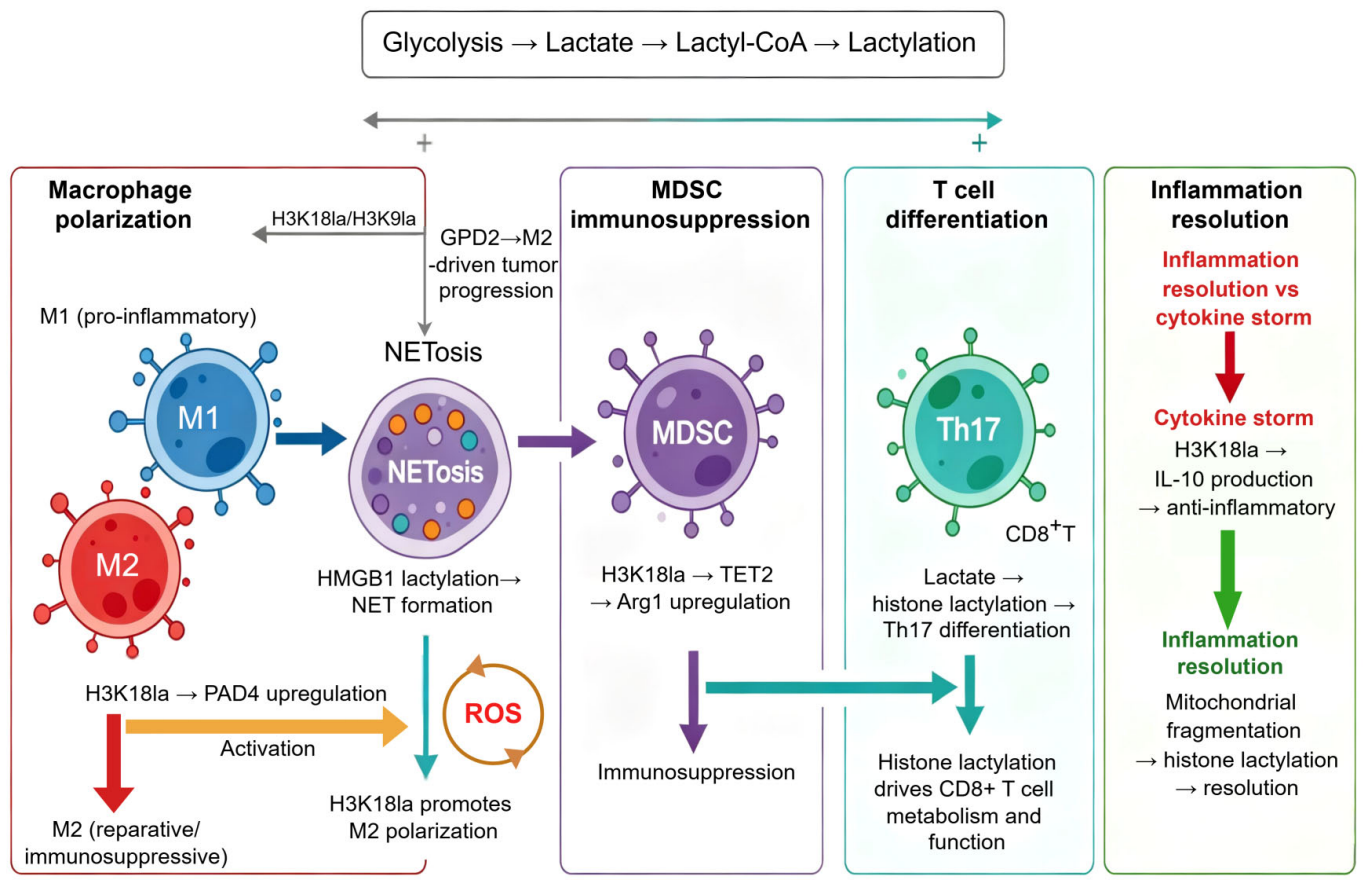
Lactylation orchestrates immune cell function and inflammation. Lactylation regulates macrophage polarization, NETosis, MDSC immunosuppression, T cell differentiation, and inflammation resolution in sepsis.

The immune system operates under stringent metabolic control, with glycolytic reprogramming serving as a hallmark of activated immune cells. Lactylation, a lactate-derived post-translational modification, has emerged as a critical epigenetic and signaling mechanism that translates metabolic flux into functional outputs across diverse immune lineages. Accumulating evidence from recent years has substantially expanded the understanding of how lactylation regulates macrophage polarization, neutrophil effector functions, myeloid-derived suppressor cell (MDSC) biology, T cell differentiation, and inflammatory resolution, with particular emphasis on context-dependent and cell-type-specific effects (**Figure 4**).

### 4.1 Macrophage polarization: M1 versus M2 phenotypes

Macrophages exhibit remarkable phenotypic plasticity, adopting pro-inflammatory (M1) or anti-inflammatory/reparative (M2) states in response to microenvironmental cues. Lactylation has been identified as a key epigenetic rheostat governing this transition. Irizarry-Caro et al. (68) demonstrated that the TLR signaling adapter BCAP promotes histone lactylation to drive the inflammatory-to-reparatory macrophage transition, establishing that lactylation facilitates phenotypic plasticity in innate immune cells. Complementing this finding, De Leo et al. (69) showed that glucose-driven histone lactylation promotes the immunosuppressive activity of monocyte-derived macrophages in glioblastoma, revealing how metabolic-epigenetic crosstalk can be co-opted to subvert immune surveillance. Mechanistically, histone lactylation at specific residues directly controls M1/M2 polarization. Irizarry-Caro et al. (12) demonstrated that BCAP promotes H3K18 lactylation at the promoters of M2-associated genes (Arg1, Mrc1), facilitating the inflammatory-to-reparatory transition. Zhang et al. (70) showed that macrophage MCT4 inhibition reduces intracellular lactate, decreasing H3K18la at pro-inflammatory gene promoters (IL-1β, IL-6) while increasing H3K18la at reparative gene promoters (IL-10, TGF-β). In sepsis, Li et al. (71) demonstrated that FGF15/FGFR4 signaling suppresses M1 polarization by inhibiting H3K18 lactylation-driven IRF7 expression, limiting TNF-α and IL-6 production. Di et al. (46) further quantified that the H3K18 lactylation-to-acetylation ratio in macrophages correlates with the balance between pro-inflammatory and anti-inflammatory cytokines in septic patients.

Recent investigations have substantially elaborated the mechanistic landscape. Chen et al. (72) demonstrated that methyl-CpG-binding protein 2 K271 lactylation inhibits atherosclerosis by promoting M2 polarization, suggesting that site-specific lactylation events can exert atheroprotective effects. Conversely, Huang et al. (73) reported that histone lactylation-driven glycerol−3−phosphate dehydrogenase 2 (GPD2) mediates M2 polarization to promote cervical cancer progression, illustrating context-dependent outcomes wherein identical polarization states produce divergent pathological consequences. Zhang et al. (70) further showed that macrophage monocarboxylate transporter 4 inhibition activates reparative genes via H3K18 lactylation to protect against atherosclerosis, identifying a specific lactylation residue linked to beneficial outcomes. The year 2025 brought additional insights: Guo et al. (74) comprehensively reviewed the bidirectional relationship between lactylation and macrophage polarization across disease contexts, emphasizing that this modification serves as a molecular hub integrating metabolic and inflammatory signals. Shu et al. (75) similarly provided an integrative perspective on lactylation in macrophages, highlighting clinical implications for inflammatory disorders. A critical insight emerging from these studies is that lactylation does not simply promote M1 or M2 polarization but rather calibrates macrophage functional states along a continuum, with the net effect determined by upstream metabolic signals and downstream transcriptional networks.

### 4.2 Neutrophil function and neutrophil extracellular trap formation

Neutrophils represent the first line of defense against invading pathogens but can also contribute to tissue damage through excessive activation and neutrophil extracellular trap (NET) formation. Emerging evidence implicates lactylation in both neutrophil recruitment and NETosis. Qiu et al. (76) identified a histone lactylation-ROS positive feedback loop that exacerbates light exposure-induced neutrophil recruitment in zebrafish, providing *in vivo* evidence that lactylation amplifies neutrophilic inflammation through redox-dependent mechanisms. Extending these observations, Zhu et al. (77) demonstrated that high mobility group box 1 (HMGB1) lactylation drives NET formation in lactate-induced acute kidney injury, linking a specific lactylated protein to pathogenic NETosis.

Recent studies have expanded the understanding of neutrophil lactylation in inflammatory diseases. Mechanistically, lactylation controls NETosis through a metabolic-epigenetic cascade. Huang et al. (78) demonstrated that p300-mediated H3K18 lactylation at the PAD4 promoter increases PAD4 expression, leading to histone citrullination, chromatin decondensation, and NET release. This pathway is driven by lactate accumulation, as inhibition of LDHA or MCT4 reduces H3K18la levels and suppresses PAD4 transcription. Zhu et al. (77) identified that HMGB1 lactylation at lysine 43 promotes its extracellular release, where extracellular HMGB1 binds to TLR4 on neutrophils to activate NETosis. Wei et al. (79) further showed that lactate-induced macrophage HMGB1 lactylation promotes NET formation, revealing intercellular crosstalk via the HMGB1-TLR4 axis. Wang et al. (80) further characterized the heterogeneity of neutrophils in neonatal sepsis based on hypoxia-glycolysis-lactylation signatures, proposing that lactylation-based molecular subtypes may refine prognostic stratification. The therapeutic potential of targeting neutrophil lactylation has been explored by Zhou et al. (81), who reported that metformin attenuates neutrophil recruitment through the H3K18 lactylation/ROS pathway, suggesting that approved medications may exert anti-inflammatory effects via modulation of this epigenetic axis. More recently, Zhou et al. (82) demonstrated that sleep deprivation activates a conserved lactate-H3K18la-RAR−related orphan receptor alpha (RORα) axis driving neutrophilic inflammation across species, providing mechanistic insight into how behavioral factors influence inflammatory responses through lactylation. Collectively, these findings position neutrophil lactylation as a promising therapeutic target, yet the specific writers and erasers governing neutrophil lactylation remain largely uncharacterized.

### 4.3 Myeloid-derived suppressor cell biology

MDSCs represent a heterogeneous population of immature myeloid cells with potent immunosuppressive functions that expand during sepsis and contribute to immunoparalysis. The role of lactylation in MDSC biology has recently gained attention. Da et al. (83) reported that histone lactylation-derived tet methylcytosine dioxygenase 2 enhances Arg1-mediated MDSC immunosuppression, providing mechanistic evidence that lactylation programs MDSC suppressive capacity. Wang et al. (84) further identified that lactylation-related gene signatures correlate with PMN-MDSC infiltration in colorectal cancer, suggesting that this modification may orchestrate MDSC accumulation in inflammatory microenvironments.

The translational relevance of MDSC lactylation is underscored by findings from Babl et al. (85), who demonstrated that lactic acid promotes an MDSC-like phenotype via HIF1α stabilization with prognostic impact in renal cell carcinoma. Liu et al. (86) recently reported that high throughput screening identified sanguinarine chloride as a multi-faceted therapeutic agent targeting lactylation-driven PMN-MDSC infiltration, offering a potential pharmacological approach to modulate MDSC function. While these studies originate primarily from cancer immunology, the conserved metabolic and epigenetic pathways suggest that analogous mechanisms operate in sepsis. Bar-Or et al. (87) comprehensively reviewed lactylation as a metabolic-epigenetic switch in sepsis, trauma, and inflammation, emphasizing that MDSC lactylation may represent a previously underappreciated driver of sepsis-induced immunosuppression. Whether MDSC lactylation contributes to the transition from hyperinflammation to immunoparalysis in septic patients remains an open question requiring dedicated investigation.

### 4.4 T cell differentiation and function

T lymphocytes undergo profound metabolic reprogramming upon activation, shifting from oxidative phosphorylation to aerobic glycolysis to support proliferation and effector functions. Lactylation has emerged as a regulator of both CD4^+^ and CD8^+^ T cell differentiation. Fan et al. (88) further established that global lactylome analysis reveals lactylation-dependent mechanisms underlying Th17 differentiation in experimental autoimmune uveitis, identifying specific lactylated proteins that orchestrate this pathogenic T cell subset. Mechanistically, lactylation controls CD4+ T cell differentiation through direct epigenetic regulation. Lopez Krol et al. (89) demonstrated that extracellular lactate is taken up via MCT1, converted to lactyl-CoA, and deposited as H3K18la at the RORγt and IL-17A promoters, promoting Th17 differentiation. Regarding CD8+ T cells, Raychaudhuri et al. (90) showed that activation under high-lactate conditions increases H3K18la and H3K9la at the promoters of effector genes (Ifng, Gzmb, Prf1), enhancing IFN-γ production, granzyme B expression, and cytotoxic capacity (90). Conversely, Wang et al. (91) reported that H3K9 lactylation in malignant cells upregulates PD-L1 and TGF-β, indirectly inducing exhaustion markers (PD-1, TIM-3) on CD8^+^ T cells.

CD8^+^ T cell biology is also subject to lactylation control. Raychaudhuri et al. (90) demonstrated that histone lactylation drives CD8^+^ T cell metabolism and function, showing that this modification supports effector differentiation and cytotoxic capacity. Sun and Chi (92) independently commented on this metabolic-epigenetic rewiring, emphasizing that lactate-dependent histone lactylation represents a previously unrecognized layer of CD8^+^ T cell regulation. Conversely, Wang et al. (91) reported that H3K9 lactylation in malignant cells facilitates CD8^+^ T cell dysfunction and poor immunotherapy response, illustrating that lactylation in non-immune cells can indirectly impair T cell immunity. Recent work by Qian et al. (93) demonstrated that histone lactylation drives METTL3 upregulation-mediated RNA m6A modification of chaperonin containing TCP1 subunit 2 (CCT2) to hinder CD8^+^ T cell survival in gastric cancer, revealing an integrated epigenetic-epitranscriptomic axis. Hao et al. (94) comprehensively reviewed lactate and lactylation as dual regulators of T-cell-mediated immunity, emphasizing that the net effect on T cell function depends on the source, magnitude, and duration of lactylation signals. This duality, in which lactylation can both support and suppress T cell function depending on cellular context, highlights the importance of distinguishing cell-autonomous from microenvironment-derived lactylation effects.

### 4.5 Regulation of cytokine storm and inflammation resolution

The cytokine storm represents a pathophysiological hallmark of sepsis-associated ARDS, and lactylation has been implicated in both the amplification and resolution of inflammatory responses. Xie et al. (95) demonstrated that circMETTL3-156aa reshapes glycolytic metabolism in macrophages to promote M1 polarization and induce cytokine storms in secondary hemophagocytic lymphohistiocytosis, establishing a direct link between lactylation and hyperinflammatory syndromes. Susser et al. (96) reported that mitochondrial fragmentation promotes inflammation resolution responses in macrophages via histone lactylation, revealing that metabolic organelle dynamics influence lactylation-dependent transcriptional programs that terminate inflammation. This finding is particularly noteworthy as it identifies a potential endogenous mechanism for restraining excessive inflammation.

The balance between pro-inflammatory and pro-resolving lactylation signals has therapeutic implications. Jiang et al. (97) demonstrated that lactate facilitates pancreatic repair following acute pancreatitis by promoting reparative macrophage polarization, suggesting that lactylation can support tissue recovery when appropriately calibrated. Similarly, Sun et al. (98) showed that lactic acid-producing probiotic Saccharomyces cerevisiae attenuates ulcerative colitis by suppressing macrophage pyroptosis, providing proof-of-concept that modulating lactate availability and downstream lactylation can ameliorate inflammatory pathology. Di et al. (46) recently proposed that the H3K18 lactylation-to-acetylation ratio may serve as a biomarker for sepsis severity, offering a quantitative framework for assessing the net inflammatory-epigenetic state in septic patients. Guo et al. (99) prospectively investigated histone lactylation in immune cells and its predictive role in sepsis progression, providing clinical evidence that lactylation levels correlate with disease trajectory. Yang et al. (100) comprehensively reviewed lactate and lactylation in immune cell function and autoimmune diseases, emphasizing that targeting the lactylation machinery holds promise for managing inflammatory disorders. A critical challenge lies in determining whether therapeutic interventions should aim to globally suppress lactylation or selectively modulate specific lactylation marks on defined substrates. The context-dependent and cell-type-specific effects of lactylation argue strongly for the latter approach, though the molecular tools to achieve such selectivity remain under development.

## 5 Lactylation in sepsis pathogenesis

Sepsis arises from a dysregulated host response to infection, driving life-threatening organ dysfunction through intertwined metabolic and immune derangements. Lactate accumulation, long regarded as a marker of tissue hypoxia, has recently been recognized as an active mediator that shapes sepsis outcomes via protein lactylation. Emerging evidence positions lactylation as a critical epigenetic node that translates glycolytic flux into pathogenic gene expression programs, immune cell dysfunction, and end-organ damage. Clinical studies have identified elevated lactylation marks in septic patients, correlating with disease severity and adverse outcomes (**Table 1**).

**Table 1:** Key clinical and preclinical studies on lactylation in sepsis pathogenesis.

Author, year	Study type/model	Sample/tissue	Lactylation mark/gene	Key findings	Ref.
Chu X, et al. (2021)	Prospective cohort	Human PBMCs	H3K18la	H3K18la elevated in septic shock, correlated with APACHE II/SOFA scores, ICU stay, MV duration, and lactate levels; AUC >0.85 for diagnosis	(13)
Huang WL, et al. (2025)	*In vivo* (CLP/LPS) + *in vitro*	Rat kidney, NRK-52E cells	H3K18la, PAD4	p300-mediated H3K18la upregulates PAD4 → activates NETosis → promotes SA-AKI; PAD4 identified as therapeutic target	(78)
Guo Y, et al. (2025)	Prospective cohort	Human immune cells	H4K5la, H3K18la	Immune cell lactylation predicted sepsis progression; combination of neutrophil H4K5la and CRP enhanced early diagnostic accuracy	(99)
Li Y, et al. (2025)	Bioinformatics + murine CLP/LPS	Human/mouse	RBM25, Acly	RBM25-Acly axis couples metabolism to histone lactylation; 5-gene signature predicted sepsis (AUC>0.85); identified novel metabolic-epigenetic circuit	(101)
Li X, et al. (2025)	Prospective observational	Human BALF	H3K18la	H3K18la independently predicted ARDS development (AUC = 0.804); levels increased on day 3 in non-survivors; correlated with SOFA scores and MV duration	(102)
Li S, et al. (2024)	Bioinformatics (RNA-seq)	Human blood	S100A11, CCNA2	Two lactylation-related genes (LRGs) identified as diagnostic/prognostic markers in sepsis	(103)
Jiang K, et al. (2025)	Transcriptomics + machine learning	Human (GSE232404)	PECR, TP53I3	118 differentially expressed LRGs identified; consensus clustering revealed two SA-AKI molecular subtypes with distinct immune profiles; PECR and TP53I3 as key LRGs in proximal tubule cells	(104)
Jin R, et al. (2026)	Integrative analysis + LPS mouse	Human (GSE79962), mouse heart	GADD45B, STAT3, SLC7A5	Three hub genes identified (AUC>0.95); linked to innate immune pathways, cGAS-STING signaling, and immune cell infiltration (NK cells, macrophages)	(105)
An S, et al. (2023)	*In vivo* (CLP/LPS) + *in vitro*	Mouse kidney	Fis1 lactylation	PDHA1 hyperacetylation → lactate overproduction → Fis1 lactylation → exacerbates SA-AKI	(106)
Qiao J, et al. (2024)	*In vivo* (CLP) + *in vitro*	Mouse kidney, HK-2 cells	H3K18la, Ezrin lactylation	Both H3K18la and Ezrin lactylation promote renal dysfunction; identified as potential therapeutic targets in SA-AKI	(108)
Luo M, et al. (2026)	Lactyl-proteomics + *in vivo*/*in vitro*	Mouse kidney	LDHB K156 lactylation	LDHB K156la links cGAS-STING-mediated metabolic reprogramming to NLRP3 inflammasome activation in SA-AKI	(109)
Yu H, et al. (2025)	*In vivo* (LPS) + *in vitro*	Mouse heart, cardiomyocytes	H3K14la	Gastrodin regulates H3K14la via CDT2-KAT2A axis to treat sepsis-induced myocardial dysfunction	(111)
Sun S, et al. (2025)	*In vivo* (LPS) + adoptive transfer	Mouse heart, monocytes	H3K18la	Exercise enhances monocyte glycolysis → lactate → p300-mediated H3K18la → restores cardiac immune homeostasis in septic cardiomyopathy	(112)
Luo S, et al. (2026)	*In vivo* (LPS/CLP) + *in vitro*	Mouse brain, neurons	AARS1-dependent lactylation	Targeting AARS1-dependent lactylation improves neuronal plasticity and mitigates cognitive deficits in sepsis-associated encephalopathy	(115)

APACHE II, Acute Physiology and Chronic Health Evaluation II; ARDS, acute respiratory distress syndrome; AUC, area under the curve; BALF, bronchoalveolar lavage fluid; cGAS, cyclic GMP−AMP synthase; CLP, cecal ligation and puncture; CRP, C−reactive protein; H3K18ac, histone H3 lysine 18 acetylation; H3K18la, histone H3 lysine 18 lactylation; H4K5la, histone H4 lysine 5 lactylation; ICU, intensive care unit; LPS, lipopolysaccharide; LRG, lactylation−related gene; MV, mechanical ventilation; NETosis, neutrophil extracellular trap formation; NK, natural killer; PBMC, peripheral blood mononuclear cell; SA−AKI, sepsis−associated acute kidney injury; SCM, septic cardiomyopathy; SOFA, Sequential Organ Failure Assessment; STING, stimulator of interferon genes.

### 5.1 Clinical evidence linking lactylation to sepsis severity

Lactylation marks are robustly associated with sepsis progression. Chu et al. (13) demonstrated that elevated histone H3K18 lactylation (H3K18la) levels in peripheral blood mononuclear cells distinguished septic shock patients from healthy controls, with area under the curve values exceeding 0.85 for diagnostic performance. Specifically, H3K18la levels in septic shock patients were approximately 2.8−fold higher than those in healthy controls (median relative intensity 1.42 vs. 0.51, P < 0.001). Furthermore, H3K18la levels correlated positively with APACHE II scores (r = 0.62, P < 0.001) and SOFA scores (r = 0.58, P < 0.001), indicating a strong association with disease severity. Extending these findings, Di et al. (101) reported that the H3K18 lactylation-to-acetylation ratio served as a superior prognostic indicator compared with lactate alone, independently predicting 28-day mortality in septic patients. The optimal cut−off value for the H3K18la/ac ratio was 0.38, with a sensitivity of 82% and specificity of 76% for predicting 28−day mortality (AUC = 0.86, 95% CI: 0.81–0.91). Patients with a ratio above this cut−off had a 3.2−fold higher risk of 28−day mortality (OR = 3.18, 95% CI: 1.95–5.18, P < 0.001). The predictive value of lactylation biomarkers has been further validated in prospective cohorts. Li et al. (102) prospectively examined H3K18la in sepsis-associated ARDS patients, demonstrating that admission lactylation levels correlated with sequential organ failure assessment scores and mechanical ventilation duration. In this cohort, patients with H3K18la levels above the median had a 2.4−fold longer median duration of mechanical ventilation (14 vs. 6 days, P = 0.008). Guo et al. (99) conducted a prospective observational study revealing that histone lactylation in circulating immune cells independently predicted sepsis progression, with higher lactylation associated with transition from hyperinflammation to immunoparalysis. Collectively, these clinical studies position lactylation marks as promising diagnostic and prognostic biomarkers, though whether elevated lactylation directly contributes to pathogenesis or merely reflects metabolic severity remains incompletely resolved. Longitudinal studies tracking lactylation dynamics throughout sepsis courses are needed to establish causal relationships.

### 5.2 Lactylation-related genes as molecular signatures

Beyond direct lactylation measurements, transcriptomic profiling of lactylation-related genes (LRGs) has emerged as a complementary approach for sepsis stratification. Li et al. (103) integrated bioinformatic analysis to identify LRG signatures that discriminated septic patients from non-infected critically ill controls, proposing a prognostic model based on nine LRGs. Jiang et al. (104) similarly explored LRG-based molecular phenotypes in sepsis-associated acute kidney injury (SA-AKI), identifying two distinct clusters with divergent immune infiltration profiles and clinical outcomes. The functional relevance of specific LRGs has been mechanistically dissected. Li et al. (101) identified the RNA−binding motif protein 25 (RBM25)-acly axis as a key regulator of lactylation in sepsis, demonstrating that RBM25 modulates ACLY expression to influence histone lactylation and inflammatory gene transcription. In septic cardiomyopathy, Jin et al. (105) employed integrative analysis to identify lactylation-associated hub genes, including pyruvate dehydrogenase kinase 1 and LDHA, which correlated with cardiac dysfunction severity. A critical limitation of current LRG studies is their reliance on bulk transcriptomics, which obscures cell-type-specific lactylation signatures. Single-cell approaches are urgently needed to resolve whether distinct immune lineages exhibit characteristic LRG expression patterns that drive their functional polarization during sepsis.

### 5.3 Lactylation in sepsis-induced organ dysfunction

Lactylation has been mechanistically implicated in multiple sepsis-associated organ injuries, with the kidney being the most extensively studied. An et al. (106) demonstrated that pyruvate dehydrogenase E1 subunit alpha 1 hyperacetylation-mediated lactate overproduction promotes SA-AKI through Fis1 (fission, mitochondrial 1) lactylation, establishing a direct link between mitochondrial metabolism and organ injury. Huang et al. (78) showed that histone lactylation-mediated PAD4 upregulation promotes SA-AKI via activating NETosis, demonstrating crosstalk between epigenetic modification and NET formation. The same group further identified an H3K18la-mediated sphingosine kinase 1-SIRT1 feedback loop that accelerates tubular epithelial cell pyroptosis in SA-AKI (107). Qiao et al. (108) provided comprehensive mechanistic evidence that both H3K18la and Ezrin lactylation promote renal dysfunction in SA-AKI, identifying these modifications as potential therapeutic targets. Luo et al. (109) recently discovered that lactate dehydrogenase B (LDHB) K156 lactylation links cyclic GMP-AMP synthase (cGAS)-STING-mediated metabolic reprogramming to NLR family pyrin domain containing 3 (NLRP3) inflammasome activation in SA-AKI, revealing an unexpected connection between lactylation and innate immune sensing pathways.

In septic cardiomyopathy, Zhong et al. (110) reviewed mitochondrial regulation of lactylation, emphasizing that organelle-specific lactylation events may drive cardiac dysfunction. Yu et al. (111) demonstrated that gastrodin regulates H3K14la through the CDT2-KAT2A axis to treat sepsis-induced myocardial dysfunction, providing proof-of-concept for pharmacological modulation of lactylation in cardiac protection. The same group further reported that jaceosidin attenuates sepsis-induced myocardial dysfunction by promoting sirtuin 2 (SIRT2)-mediated inhibition of H3K18la (111). Sun et al. (112) demonstrated that exercise-induced histone lactylation in monocyte-derived macrophages restores cardiac immune homeostasis in sepsis-induced cardiomyopathy, suggesting that physiological stimuli that modulate lactylation may confer therapeutic benefit. In sepsis-associated acute lung injury, Lu et al. (23) showed that lactylation of histone H3K18 and early growth response protein 1 promotes endothelial glycocalyx degradation, providing a mechanistic link between lactylation and vascular barrier dysfunction. Despite this progress, most studies focus on individual organs, and whether lactylation drives multi-organ failure through shared or organ-specific mechanisms remains unknown. Comparative proteomic analyses across organs from the same septic animals are needed to address this gap.

### 5.4 Preclinical evidence for therapeutic targeting

Preclinical studies have begun evaluating strategies to modulate lactylation in sepsis models. Luo et al. (113) demonstrated that curcumin intervention ameliorates SA-AKI by regulating p300 expression and protein lactylation, suggesting that natural products may exert renoprotective effects through lactylation modulation. Mokhtari et al. (114) reported that mitotherapy attenuates sepsis-induced brain injury in cecal ligation and puncture rats, with effects mediated in part through the SIRT1-peroxisome proliferator−activated receptor gamma coactivator 1−alpha network that influences lactylation dynamics. In septic cardiomyopathy, Yu et al. (111) showed that pharmacological inhibition of KAT2A reduced H3K14la levels and improved cardiac function. Luo et al. (115) provided compelling evidence that targeting AARS1-dependent lactylation improves neuronal process plasticity and mitigates cognitive deficits in sepsis-associated encephalopathy, identifying AARS1 as a tractable therapeutic target. These preclinical studies collectively demonstrate that lactylation is pharmacologically modifiable, but several challenges remain. First, the specificity of currently available inhibitors for lactylation versus acetylation is poorly characterized. Second, systemic inhibition of writers or activation of erasers may disrupt physiological lactylation required for normal immune homeostasis. Third, most studies have evaluated interventions at the time of sepsis induction, whereas patients typically present later in the disease course. Testing therapeutic windows in clinically relevant delayed-intervention models is essential before clinical translation. A comprehensive overview of notable research on lactylation in sepsis and ARDS, including clinical biomarkers, mechanistic pathways, and therapeutic implications, is provided in **Table 2**.

**Table 2:** Overview of notable research on lactylation in sepsis and ARDS.

Context	Key findings	Implications	Ref.
Clinical biomarker	Elevated H3K18la in PBMCs distinguishes septic shock from healthy controls (AUC >0.85); correlates with APACHE II/SOFA scores, ICU stay, MV duration, and lactate levels.	H3K18la is a potential diagnostic and prognostic biomarker for sepsis severity.	(13)
Clinical biomarker	H3K18 lactylation-to-acetylation ratio independently predicts 28-day mortality and outperforms lactate alone.	The ratio offers a quantitative framework for assessing inflammatory-epigenetic state.	(49)
Neutrophil NETosis	p300-mediated H3K18la at PAD4 promoter increases PAD4 expression, leading to histone citrullination, chromatin decondensation, and NET release.	Targeting H3K18la-PAD4 axis may attenuate NET-driven organ injury in sepsis.	(81)
Neutrophil inflammation	Glycolysis-derived lactate drives H4K8la at WTAP promoter, upregulating WTAP and m6A-mediated TLR2 mRNA stabilization, causing neutrophil hyperinflammation.	Identifies an integrated epigenetic-epitranscriptomic cascade as a therapeutic target.	(126)
Macrophage polarization	BCAP promotes H3K18la at M2 gene promoters (Arg1, Mrc1), driving inflammatory-to-reparatory transition.	Lactylation facilitates phenotypic plasticity and resolution of inflammation.	(12)
Macrophage polarization	FGF15/FGFR4 signaling suppresses M1 polarization by inhibiting H3K18la-driven IRF7 expression, limiting TNF-α and IL-6 in sepsis.	Endogenous pathway that can be therapeutically engaged to control hyperinflammation.	(74)
Alveolar epithelial apoptosis	Lactate drives H3K18la at caspase-8 promoter, upregulating caspase-8 and promoting TNF-α-induced AEC apoptosis in sepsis-induced lung injury.	H3K18la-caspase-8 axis as a potential target to limit epithelial injury.	(125)
Endothelial ferroptosis	Glycolysis-derived lactate promotes H3K14la at TFRC/SLC40A1 promoters, driving endothelial ferroptosis and vascular dysfunction in sepsis-induced ARDS.	H3K14la-ferroptosis axis is a novel mechanistic link for endothelial injury.	(120)
Endothelial glycocalyx degradation	H3K18la and Egr1 lactylation promote heparanase/syndecan-1 expression, degrading glycocalyx and exacerbating lung injury.	Lung-selective mechanism that may be targeted to preserve vascular barrier function.	(26)
Alveolar epithelial ferroptosis	PDK4-driven lactate accumulation facilitates AARS1-mediated LPCAT2 K375la, suppressing STAT1 acetylation and SLC7A11, leading to epithelial ferroptosis.	PDK4-LPCAT2 lactylation axis as a therapeutic node for SI-ALI.	(133)
Autophagy and immunosuppression	H3K18la promotes autophagic gene expression to mitigate immunosuppression in sepsis.	Complete ablation of lactylation may be harmful; timing matters for therapy.	(25)
Sepsis-associated encephalopathy	AARS1-dependent lactylation of neuronal proteins affects process plasticity and cognitive function.	Targeting AARS1 lactylation improves neurological outcomes in sepsis.	(118)
Septic cardiomyopathy	Exercise-induced histone lactylation in monocyte-derived macrophages restores cardiac immune homeostasis.	Physiological stimuli that modulate lactylation may confer therapeutic benefit.	(115)

AEC, alveolar epithelial cell; APACHE II, Acute Physiology and Chronic Health Evaluation II; AUC, area under the curve; ICU, intensive care unit; MDSC, myeloid−derived suppressor cell; MV, mechanical ventilation; NET, neutrophil extracellular trap; PBMC, peripheral blood mononuclear cell; SA−AKI, sepsis−associated acute kidney injury; SI−ALI, sepsis−induced acute lung injury; SOFA, Sequential Organ Failure Assessment.

## 6 Lactylation in sepsis-associated ARDS: mechanistic insights

Sepsis-associated ARDS represents a severe pulmonary complication of systemic infection, driven by glycolytic reprogramming and excessive inflammatory responses. Emerging evidence implicates lactylation as a critical epigenetic mechanism bridging metabolic derangements with lung injury pathogenesis. Recent studies have dissected specific lactylation-mediated pathways controlling endothelial dysfunction, alveolar epithelial cell fate, and immune cell infiltration in the injured lung (**Table 3**).

**Table 3:** Key mechanistic studies of lactylation in sepsis-associated ARDS.

Author, year	Model system	Lactylation type	Molecular mechanism	Cell type/tissue	Key finding	Ref.
Du B et al., 2026	LPS-stimulated neutrophils; septic mouse model; pediatric sepsis patients	H4K8la (histone)	Glycolysis → H4K8la enrichment at WTAP promoter → WTAP transcription → m6A-mediated TLR2 mRNA stabilization → TLR2 upregulation → neutrophil hyperinflammation	Neutrophils	Glycolysis/H4K8la/WTAP/m6A/TLR2 cascade drives neutrophil overinflammation in sepsis	(123)
Gong F et al., 2025	CLP mouse model; LPS-stimulated ECs	H3K14la (histone)	Glycolysis-derived lactate → H3K14la enrichment at TFRC/SLC40A1 promoters → EC ferroptosis	Pulmonary endothelial cells	Glycolysis/H3K14la/ferroptosis axis drives vascular dysfunction in sepsis-induced ARDS	(117)
Wang Y et al., 2025	Blood transcriptome from sepsis-associated ARDS patients	Lactylation activity score (based on LRGs)	High-lactylation phenotype vs. low-lactylation phenotype; six key markers (ALDOB, CCT5, EP300, PFKP, PPIA, SIRT1) differentiate phenotypes	Circulating immune cells	High-lactylation activity correlates with longer hospital stay, higher mortality, and distinct immune profiles	(119)
Suo T et al., 2025	Multi-cohort transcriptomics; CLP/LPS murine models	Lactylation-related gene signature (ALDH1A1 hub gene)	ALDH1A1 downregulation correlates with histone lactylation levels; ALDH1A1-associated MDSC and neutrophil infiltration	Immune cells in lung tissue	Protein-verified LRG signature with diagnostic relevance; transcriptional–translational discordance identified	(120)
Lin P et al., 2026	CLP mouse model; LPS-stimulated AECs	H3K18la (histone)	Lactate → H3K18la enrichment at caspase-8 promoter → caspase-8 upregulation → TNF-α-induced AEC apoptosis	Alveolar epithelial cells	Lactate/H3K18la/caspase-8 axis promotes AEC apoptosis in sepsis-induced lung injury	(122)
Deng Y et al., 2025	CLP mouse model; LPS-stimulated epithelial cells	LPCAT2 K375la (non-histone)	PDK4-driven glycolysis → lactate accumulation → AARS1-mediated LPCAT2 K375la → suppresses STAT1 acetylation → SLC7A11 repression → epithelial ferroptosis	Alveolar epithelial cells	PDK4/LPCAT2 lactylation/ferroptosis axis exacerbates SI-ALI	(126)
Chen L et al., 2026	CLP mouse model; LPS-stimulated macrophages	Histone lactylation (H3K18la regulation)	Pharmacological elevation of lactate → H3K18la-mediated IL-10 production → anti-inflammatory effects	Macrophages	Controlled lactylation modulation exerts anti-inflammatory effects in sepsis	(127)
Xie J et al., 2026	LPS-induced ALI mouse model; M1 macrophages	Histone lactylation (indirect regulation)	HIF-1α-mediated glycolysis → histone lactylation → confers ferroptosis resistance in M1 macrophages	M1 macrophages	Lactylation promotes adaptive responses that limit tissue damage during ALI	(128)

AEC, alveolar epithelial cell; ALI, acute lung injury; ARDS, acute respiratory distress syndrome; CLP, cecal ligation and puncture; EC, endothelial cell; LRG, lactylation-related gene; MDSC, myeloid-derived suppressor cell; PMVEC, pulmonary microvascular endothelial cell; SI-ALI, sepsis-induced acute lung injury; S-ALI, sepsis-induced acute lung injury. Note: Studies cited include both sepsis models (CLP, cecal ligation and puncture) and direct lung injury models (LPS−i.t., intratracheal lipopolysaccharide), which mimic pneumonia−induced ARDS. The consistency of findings across models suggests that lactylation mechanisms may be conserved across different ARDS etiologies.

### 6.1 The glycolysis-lactylation-ferroptosis axis

A growing body of evidence links glycolysis-driven lactylation to ferroptotic cell death in sepsis-induced lung injury. Gong et al. (116) identified a novel pathway wherein lactic acidemia promotes pulmonary endothelial cell death through the cold-inducible RNA-binding protein (CIRP)-Z−DNA binding protein 1 (ZBP1) -PANoptosis axis, establishing that lactate accumulation directly engages cell death machinery in the pulmonary vasculature. More specifically, Gong et al. (117) demonstrated that histone H3K14 lactylation drives endothelial dysfunction in sepsis-induced ARDS by promoting solute carrier family 40 member 1 (SLC40A1) and transferrin-mediated ferroptosis, revealing a direct molecular link between epigenetic lactylation and iron-dependent cell death. This finding aligns with observations from Qin et al. (118), who reported that Sirtuin 3 attenuates acute lung injury by decreasing both ferroptosis and inflammation through inhibition of aerobic glycolysis, suggesting that delactylation mechanisms may counteract pro-ferroptotic signals. The convergence of these studies indicates that lactylation operates as a rheostat controlling ferroptotic sensitivity in pulmonary endothelium. A critical unresolved question concerns whether lactylation promotes ferroptosis directly through transcriptional regulation of iron-handling genes or indirectly via metabolic reprogramming that primes cells for ferroptotic execution. Distinct lactylation marks on histone versus non-histone substrates may differentially contribute to these outcomes, necessitating site-specific mechanistic dissection.

### 6.2 Lactylation-based phenotypes and molecular signatures

Clinical heterogeneity in sepsis-associated ARDS has prompted efforts to stratify patients using lactylation-based molecular phenotyping. Wang et al. (119) characterized lactylation-based phenotypes in sepsis-associated ARDS, identifying high-lactylation and low-lactylation activity clusters that exhibited distinct clinical outcomes and immune profiles. Patients with elevated lactylation activity demonstrated higher sequential organ failure assessment scores, prolonged mechanical ventilation requirements, and increased 28-day mortality compared with their low-lactylation counterparts. Complementing this phenotypic stratification, Suo et al. (120) employed multi-omics integration with machine learning to identify lactylation-related hub genes in SA-ARDS, including aldehyde dehydrogenase 1 family member A1 (ALDH1A1), calmodulin 1 (CALM1), cyclin A2 (CCNA2), histone cluster 1 H2B family member N (HIST1H2BN), and SH3 domain containing GRB2 like 1 (SH3GL1), which collectively discriminated ARDS patients from septic controls with high diagnostic accuracy. The functional relevance of these signatures was experimentally validated, revealing that ALDH1A1 expression inversely correlated with histone lactylation levels in pulmonary immune cells. Extending these observations to septic cardiomyopathy, Jin et al. (105) similarly identified lactylation-associated hub genes, suggesting that lactylation-based molecular signatures may have diagnostic utility across sepsis-induced organ dysfunction syndromes. Qian et al. (121). further employed integrative bioinformatics and machine learning to identify lactylation-related biomarkers in sepsis, validating their prognostic performance in independent cohorts. Collectively, these studies position lactylation-based molecular phenotyping as a promising strategy for patient stratification, though prospective validation in multicenter cohorts is required before clinical implementation.

### 6.3 Lactylation in alveolar epithelial cell fate

Beyond endothelial dysfunction, lactylation directly influences alveolar epithelial cell survival and function during sepsis. Lin et al. (122) demonstrated that histone H3 lysine 18 lactylation promotes alveolar epithelial cell apoptosis in sepsis-induced lung injury by upregulating caspase-8 expression, establishing a direct mechanistic link between lactylation and epithelial cell death. This pro-apoptotic effect was validated both *in vivo* and *in vitro*, with H3K18la enrichment observed at the caspase-8 promoter in injured lung tissue. Specifically, Yang et al. (22) found that H3K18la enhanced autophagic flux in immune cells, which paradoxically limited excessive inflammation and improved survival in septic models. The apparent discrepancy between pro-apoptotic effects in epithelial cells and pro-autophagic effects in immune cells highlights the importance of cell-type-specific lactylation responses. A critical insight emerging from these contrasting findings is that therapeutic modulation of lactylation must consider cell lineage selectivity. Global manipulation of lactylation may produce opposing effects across different pulmonary compartments, arguing for cell-targeted approaches.

### 6.4 Lactylation and immune cell infiltration in ARDS

Immune cell infiltration into the alveolar space represents a pathological hallmark of SA-ARDS, and emerging evidence implicates lactylation in recruiting and programming infiltrating myeloid cells. Suo et al. (120) demonstrated that the lactylation-related hub gene ALDH1A1 correlated significantly with infiltration of myeloid-derived suppressor cells (MDSCs) and neutrophils in ARDS lung tissue, as determined by single-sample gene set enrichment analysis and Cell−type Identification by Estimating Relative Subsets of RNA Transcripts deconvolution. This observation was experimentally confirmed, showing that ALDH1A1 knockdown reduced histone lactylation levels and attenuated chemokine-driven neutrophil recruitment. Du et al. (123) reported that upregulation of the N_6_−methyladenosine writer WTAP by histone lactylation promotes inflammatory responses via TLR2 in neutrophils, revealing an integrated epigenetic-epitranscriptomic axis that amplifies neutrophilic inflammation in sepsis. Bar-Or et al. (87) comprehensively reviewed lactylation as a metabolic-epigenetic switch in sepsis and inflammation, emphasizing that lactylation-dependent immune cell infiltration represents a conserved mechanism across multiple inflammatory conditions. A notable contrast emerges between the pro-inflammatory role of lactylation in neutrophil recruitment and its potential anti-inflammatory effects in other contexts. This functional duality suggests that lactylation effects on immune infiltration are highly dependent on the specific immune subset, the timing of lactylation signals, and the local metabolic microenvironment.

### 6.5 Lactylation as a metabolic-epigenetic bridge in ARDS

Synthesizing the available evidence, lactylation functions as an integrative bridge connecting metabolic reprogramming to immune dysregulation in SA-ARDS. Zhang et al. (124) comprehensively reviewed how lactate metabolic reprogramming drives histone lactylation to orchestrate immune cell fate decisions in sepsis, emphasizing that this modification translates glycolytic flux into durable transcriptional changes that shape inflammatory outcomes. The metabolic-epigenetic linkage is bidirectional: glycolysis fuels lactylation, which in turn reinforces glycolytic gene expression through positive feedback mechanisms. Li et al. (125) developed a novel prognostic model for sepsis patients based on dysregulated immune cell lactylation, providing clinical evidence that lactylation signatures in peripheral immune cells reflect systemic metabolic-inflammatory states. Deng et al. (126) discovered that pyruvate dehydrogenase kinase 4 (PDK4)-driven lactate accumulation facilitates lysophosphatidylcholine acyltransferase 2 (LPCAT2) lactylation to exacerbate sepsis-induced acute lung injury, revealing that non-histone lactylation of metabolic enzymes can amplify pathogenic cascades. Therapeutic proof-of-concept has emerged from multiple studies. Chen et al. (127) demonstrated that pharmacological elevation of lactate alleviates sepsis via histone lactylation-induced IL-10 production, suggesting that controlled modulation of lactylation may exert anti-inflammatory effects. Xie et al. (128) reported that HIF-1α-mediated lactate metabolism confers ferroptosis resistance in M1 macrophages through histone lactylation during acute lung injury, indicating that lactylation may also promote adaptive responses that limit tissue damage. Collectively, these findings position lactylation as a nodal point integrating metabolic cues, epigenetic regulation, and immune effector functions in SA-ARDS. A major challenge lies in distinguishing causal contributions of lactylation from correlative metabolic changes, which will require genetic tools that specifically ablate lactylation without disrupting global lactate metabolism.

### 6.6 Organ-specificity of lactylation in sepsis

The multi-organ dysfunction characteristic of sepsis raises the critical question of whether lactylation exhibits organ-specific patterns and pathophysiological roles. In the lung, lactylation directly contributes to the endothelial injury and glycocalyx degradation that define ARDS. Lu et al. (23) demonstrated that H3K18 lactylation and early growth response protein 1 lactylation promote endothelial glycocalyx degradation in sepsis-induced acute lung injury, providing a direct mechanistic link between this epigenetic mark and vascular barrier dysfunction. Specifically, lactate drives H3K18la enrichment at the promoters of heparanase and syndecan-1, enzymes that degrade glycocalyx components. This finding is lung-selective to date and has not been reported in other organs. Organ-specific alterations in lactylation patterns are evident when comparing the lung, kidney, heart, and brain during sepsis. In the kidney (SA-AKI), lactylation predominantly activates NETosis pathways (H3K18la-PAD4) and tubular epithelial cell pyroptosis (H3K18la-SPHK1-SIRT1 feedback loop) (78, 107, 108). In the heart (septic cardiomyopathy), lactylation regulates metabolic enzymes and mitochondrial function, with H3K14la and H3K18la modulating fatty acid oxidation and autophagy (111, 112). In the brain (sepsis-associated encephalopathy), AARS1-dependent lactylation of neuronal proteins affects process plasticity and cognitive function (115). In the lung, beyond endothelial injury, lactylation also controls alveolar epithelial cell apoptosis via H3K18la-caspase-8 axis and ferroptosis via H3K14la-SLC40A1/transferrin pathway (117, 122).

Lactylation exerts distinct effects on immune versus non−immune cells in the septic lung. In immune cells, lactylation primarily regulates inflammatory activation and polarization. For example, H3K18la promotes M2 macrophage polarization (12) and suppresses M1 via FGF15/FGFR4 signaling (71); it drives NETosis in neutrophils (77, 78), Th17 differentiation in CD4^+^ T cells (89), and effector function in CD8+ T cells (90). In contrast, non−immune cells (alveolar epithelial and endothelial cells) respond to lactylation mainly through altered survival and barrier integrity. H3K18la induces alveolar epithelial apoptosis (122) (whereas the same mark promotes autophagic survival in immune cells), and endothelial H3K14la drives ferroptosis (117) while H3K18la/Egr1 lactylation degrades glycocalyx. Non−histone ENO1 lactylation further disrupts endothelial barrier function (129, 130). This functional dichotomy suggests that global lactylation modulation may produce opposing effects, underscoring the need for cell−type−specific or timed interventions (119).

Beyond histone lactylation, non−histone lactylation of the glycolytic enzyme enolase 1 (ENO1) has recently emerged as a critical mechanism in pulmonary endothelial dysfunction during ARDS and sepsis. A study demonstrated that hyperlactylation at lysine 193 (K193) of ENO1 in pulmonary endothelial cells drives CXCL12 expression by relieving ENO1−mediated translational suppression of CXCL12 mRNA, thereby promoting chemokine release and endothelial injury (131). Notably, K193 hyperlactylation also enhances ENO1 enzymatic activity, creating a positive feedback loop that amplifies glycolysis and sustains lactylation. Suppression of lactate−induced lactylation mitigated ARDS development (131). Complementing this finding, a study showed that P300−mediated lactylation of ENO1 at lysine 71 (K71) in sepsis reduces ENO1 binding to TRIM21 mRNA, preventing its degradation and leading to TRIM21 upregulation. Elevated TRIM21 then promotes ubiquitination and degradation of vascular endothelial−cadherin (VE−Cadherin), disrupting endothelial adherens junctions and increasing permeability (132). Targeting K71 lactylation with a specific inhibitory peptide alleviated endothelial injury and improved survival in septic mice (132). Together, these studies establish ENO1 lactylation as a key non−histone mechanism linking glycolytic flux to endothelial barrier dysfunction in the septic lung, complementing the histone lactylation pathways described above.

Direct comparative studies of lactylation across lung, liver, kidney, and heart from the same septic animals are lacking. However, existing evidence suggests that lactylation patterns are shaped by organ-specific metabolic baselines. For instance, the liver, which expresses high levels of LDHA and MCT4, may exhibit distinct lactylation kinetics compared with the kidney, but no dedicated hepatic studies exist in sepsis models. The heart, with its high oxidative capacity, may show delayed lactylation responses relative to the lung, which is directly exposed to circulating lactate from inflamed organs. Future research should prioritize multi-organ lactylome profiling from the same septic animals to resolve these organ-specific signatures and identify whether common or distinct lactylation pathways drive multi-organ failure.

### 6.7 Lactylation in non−sepsis ARDS

Although sepsis is the leading cause of ARDS, pneumonia−induced ARDS accounts for a substantial proportion of cases in intensive care units. Several studies have investigated lactylation in acute lung injury (ALI) models that mimic direct pulmonary insults, including intratracheal lipopolysaccharide (LPS) administration, which models pneumonia−induced ARDS. Xie et al. (128) used an LPS−induced ALI mouse model (LPS administered intratracheally) and demonstrated that HIF−1α−mediated glycolysis drives histone lactylation, which in turn confers ferroptosis resistance in M1 macrophages, representing an adaptive response that limits tissue damage. This model directly reflects lung−specific injury rather than systemic sepsis, and the findings suggest that lactylation plays a protective role in pneumonia−associated lung injury. Chen et al. (127) utilized both CLP (cecal ligation and puncture, a sepsis model) and intratracheal LPS models to show that pharmacological elevation of lactate alleviates lung injury via histone lactylation−induced IL−10 production. The consistency of findings across sepsis and direct LPS−induced ALI models indicates that the metabolic−epigenetic axis of lactylation operates in both systemic and local lung inflammatory settings. In the LPS−i.t. model, lactate accumulation is derived from activated alveolar macrophages and infiltrating neutrophils rather than systemic circulation, yet the downstream effects on histone lactylation and gene expression are similar.

Direct comparative studies of lactylation between sepsis−associated ARDS and pneumonia−induced ARDS are lacking. However, existing evidence suggests that the core mechanisms, including H3K18la−driven NETosis, H3K14la−mediated ferroptosis, and H3K18la−induced autophagic gene expression, may be conserved across different ARDS etiologies, as they have been validated in both CLP and LPS−i.t. models (61, 78, 117, 126). Future research should systematically compare lactylation patterns in lung tissues from patients with ARDS of different causes (sepsis, pneumonia, aspiration, trauma) to identify etiology−specific or common signatures.

## 7 Therapeutic potential and clinical translation

The emerging recognition of lactylation as a critical epigenetic driver in sepsis-associated ARDS has spurred interest in therapeutic strategies targeting this modification. Preclinical evidence demonstrates that pharmacological modulation of lactate production, writer/eraser enzymes, and lactylation-reading events can attenuate organ injury and improve outcomes in sepsis models. However, translating these findings into clinical practice requires overcoming substantial challenges, including off-target effects, substrate competition with acetylation, and the context-dependent duality of lactylation functions (**Table 4**). Nevertheless, lactylation-based molecular phenotyping offers promise for patient stratification and precision medicine approaches in sepsis-associated ARDS.

**Table 4:** Summary of preclinical therapeutic strategies targeting lactylation in sepsis-associated organ dysfunction.

Author, year	Intervention/agent	Target/enzyme	Mechanism of action	Sepsis model/organ	Primary effect	Ref.
Yu H et al., 2025	Gastrodin (natural product)	CDT2-KAT2A (writer) axis	Enhances CDT2-KAT2A binding, promotes ubiquitin-mediated KAT2A degradation, reduces H3K14la	LPS-induced SIMD/heart	Reduces myocardial injury, serum cTnT/CK-MB, and pro-inflammatory cytokines	(111)
Sun S et al., 2025	Exercise (physiological intervention)	p300 (writer)/HDAC2 (eraser)	Enhances monocyte glycolysis, increases lactate production, drives H3K18la; accelerates pro-reparative macrophage transition	CLP and LPS-induced SICM/heart	Restores cardiac immune homeostasis and function; adoptive transfer of lactylated monocytes improves outcomes	(112)
Luo M et al., 2025	Curcumin (natural product)	p300 (writer)	Inhibits p300 expression and decreases protein lactylation	CLP-induced SA-AKI/kidney	Ameliorates renal tubular injury, inflammation, and oxidative stress	(113)
Deng Y et al., 2026	PDK4 inhibition (metabolic targeting)	PDK4/LPCAT2 (non-histone)	Reduces PDK4-driven lactate accumulation; decreases LPCAT2 lactylation via AARS1	SI-ALI/lung	Attenuates acute lung injury and reduces pulmonary inflammation	(126)
Li Z et al., 2026	PROTAC JPS016	Class I HDACs (erasers)	Degrades Class I HDACs via PROTAC; activates PINK1/Parkin mitophagy and exopher formation; increases histone lactylation	LPS-induced SCM/heart	Enhances mitochondrial quality control, reduces cardiac injury	(136)
Yu H et al., 2026	Jaceosidin (natural product)	SIRT2 (eraser)	Activates SIRT2, specifically inhibits H3K18la; modulates IL-6, BAX and BCL-2 expression	LPS-induced SIMD/heart	Improves cardiac function (LVEF/LVFS) and reduces myocardial lactylation	(129)

CLP, cecal ligation and puncture; H3K14la, histone H3 lysine 14 lactylation; H3K18la, histone H3 lysine 18 lactylation; HDAC, histone deacetylase; LPS, lipopolysaccharide; LVEF, left ventricular ejection fraction; LVFS, left ventricular fractional shortening; PDK4, pyruvate dehydrogenase kinase 4; PROTAC, proteolysis targeting chimera; SA−AKI, sepsis−associated acute kidney injury; SAE, sepsis−associated encephalopathy; SCM, septic cardiomyopathy; SI−ALI, sepsis−induced acute lung injury; SICM, sepsis−induced cardiomyopathy; SIMD, sepsis−induced myocardial dysfunction.

### 7.1 Targeting lactate production and glycolysis

Intervening at the level of lactate generation represents the most proximal approach to modulating lactylation. Glycolysis inhibitors such as 2-deoxyglucose and oxamate have been evaluated in preclinical sepsis models, with studies demonstrating that reducing glycolytic flux attenuates histone lactylation and downstream inflammatory gene expression (133, 134). However, broad inhibition of glycolysis raises concerns regarding metabolic toxicity, as activated immune cells require aerobic glycolysis for effector functions. Yang et al. (135) demonstrated that lactate promotes macrophage HMGB1 lactylation and exosomal release in polymicrobial sepsis, suggesting that targeting lactate transport or metabolism may offer more selective intervention. A critical distinction must be drawn between inhibiting lactate production globally versus blocking its conversion to lactyl-CoA, with the latter strategy potentially preserving metabolic homeostasis while abrogating pathogenic epigenetic signaling.

### 7.2 Modulating lactylation writers and erasers

The enzymatic machinery governing lactylation deposition and removal presents tractable pharmacological targets. p300, initially identified as a lactyltransferase by Zhang et al., can be inhibited by small molecules such as curcumin, which Luo et al. (113) showed ameliorates sepsis-associated acute kidney injury through regulation of p300 expression and protein lactylation. Conversely, activating delactylases represents an alternative strategy. Du et al. (77) demonstrated that SIRT1 and SIRT3 function as robust lysine delactylases, and their activation may counteract pathogenic hyperlactylation. Of particular translational interest, Li et al. (71) reported that fibroblast growth factor 15 (FGF15)/fibroblast growth factor receptor 4 (FGFR4) signaling suppresses M1 macrophage polarization by inhibiting H3K18 lactylation-driven interferon regulatory factor 7 expression, identifying an endogenous pathway that can be therapeutically engaged. The dual functionality of Class I HDACs as both deacetylases and delactylases complicates therapeutic targeting, as inhibitors may simultaneously affect both modifications. proteolysis targeting chimera (PROTAC) -mediated degradation of Class I HDACs, as recently reported by Li and Zhang (136), offers enhanced specificity but requires further validation in sepsis models.

### 7.3 Lactylation-based diagnostic and prognostic biomarkers

The association between lactylation marks and clinical outcomes supports their utility as biomarkers. Chu et al. (13) first demonstrated that elevated H3K18 lactylation levels in peripheral blood mononuclear cells distinguished septic shock patients from healthy controls. Wang et al. (119) subsequently characterized lactylation-based phenotypes in sepsis-associated ARDS, identifying high- and low-lactylation activity clusters with divergent 28-day mortality. Sun et al. (137) comprehensively reviewed the potential of lactylation as a diagnostic and therapeutic biomarker in sepsis, emphasizing that tissue-specific lactylation patterns may guide intervention timing. However, a critical limitation remains: most studies have examined lactylation in bulk peripheral blood cells, whereas organ-specific lactylation signatures may better reflect disease pathogenesis. The development of circulating lactylation-based liquid biopsies represents a promising but largely unexplored direction.

### 7.4 Pharmacological and natural product interventions

Multiple pharmacological agents have demonstrated efficacy in sepsis models through lactylation modulation. Yu et al. (111, 129) showed that gastrodin regulates H3K14la via the CDT2-KAT2A axis to treat sepsis-induced myocardial dysfunction, while the same group reported that jaceosidin attenuates cardiac dysfunction by promoting SIRT2-mediated inhibition of H3K18 lactylation. Notably, exercise-induced histone lactylation in monocyte-derived macrophages restored cardiac immune homeostasis in sepsis-induced cardiomyopathy, suggesting that physiological stimuli that modulate lactylation may confer therapeutic benefit (112). Deng et al. (126) demonstrated that PDK4-driven lactate accumulation facilitates LPCAT2 lactylation to exacerbate sepsis-induced acute lung injury, identifying PDK4 as a potential upstream therapeutic node. Despite these promising preclinical findings, the specificity of currently available agents for lactylation versus acetylation remains poorly characterized, and most studies have not evaluated therapeutic windows relevant to clinical presentation.

### 7.5 Challenges and future directions for clinical translation

Several obstacles must be overcome before lactylation−targeted therapies reach clinical trials. First, the extensive crosstalk between lactylation and acetylation, mediated by shared writer and eraser enzymes, raises concerns that targeting these enzymes may produce unintended epigenetic consequences. Second, lactylation exerts context-dependent and cell-type-specific effects, promoting ferroptosis in pulmonary endothelium while supporting autophagic survival in immune cells, suggesting that global modulation may be counterproductive (22, 61, 117). Third, the optimal therapeutic window remains undefined; Yang et al. (22) demonstrated that H3K18 lactylation promotes autophagic gene expression to mitigate immunosuppression in sepsis, indicating that complete ablation of lactylation may be harmful. Future directions should prioritize development of substrate-selective inhibitors, cell-type-specific delivery systems, and validated pharmacodynamic biomarkers to guide patient selection and dosing. The integration of lactylation-based molecular phenotyping with machine learning approaches, as proposed by Wang et al. (119), may enable precision targeting of high-lactylation phenotypes most likely to benefit from intervention.

Despite the promising preclinical landscape, several critical limitations temper the therapeutic optimism. First, the extensive crosstalk between lactylation and acetylation, mediated by shared writers (p300) and erasers (HDAC1-3), raises specificity concerns. Pharmacological inhibition of p300 or HDACs will simultaneously affect both modifications, making it difficult to attribute therapeutic effects solely to lactylation modulation (47, 48). Second, lactylation exerts opposing, context-dependent effects: it promotes ferroptosis in pulmonary endothelium (117) while supporting autophagic survival in immune cells (22); it drives NETosis in neutrophils yet facilitates inflammation resolution in macrophages (78). Global modulation of lactylation may therefore produce unintended pro-inflammatory or pro-injury effects depending on cell type and disease phase. Third, the optimal therapeutic window remains undefined. During the hyperinflammatory phase, excessive lactylation drives organ injury, whereas during the subsequent immunoparalysis phase, lactylation promotes autophagic gene expression to mitigate immunosuppression (22). Complete ablation of lactylation may thus be harmful in later stages. Fourth, most preclinical studies have evaluated interventions at the time of sepsis induction, whereas patients typically present later in the disease course, limiting translational relevance. Fifth, no selective lactylation inhibitors or activators are clinically available; curcumin and gastrodin have broad specificity (111, 113), and PROTACs targeting HDACs remain experimental (136). Finally, the safety of modulating a ubiquitous metabolic-epigenetic pathway during pregnancy or in patients with hepatic or renal impairment has not been assessed. These constraints underscore that targeting lactylation, while promising, requires careful consideration of timing, cell-type selectivity, and off-target effects before clinical translation.

## 8 Future perspectives

Lactylation is an epigenetic bridge connecting metabolic reprogramming to immune dysregulation in sepsis−associated ARDS, but several knowledge gaps remain before clinical translation. Recent advances have begun illuminating the mechanistic landscape, but the field now faces the challenge of moving from descriptive correlative studies to causal mechanistic dissection and therapeutic validation.

A fundamental unresolved question concerns the tissue-specificity of lactylation responses during sepsis. While bulk measurements in peripheral blood have revealed associations between lactylation marks and disease severity (125, 137), whether organ-specific lactylation signatures drive distinct pathological outcomes remains unknown. Comparative proteomic analyses across lung, kidney, heart, and liver from the same septic animals are urgently needed to resolve this gap. Furthermore, the extensive crosstalk between lactylation and acetylation, mediated by shared writer enzymes such as p300 and erasers including HDAC1-3, raises important questions about substrate competition (47, 48). Current analytical methods cannot simultaneously quantify multiple acyl modifications at single-lysine resolution, limiting the ability to determine whether observed functional effects arise specifically from lactylation rather than from concomitant changes in acetylation or other modifications. Developing advanced mass spectrometry approaches capable of resolving this combinatorial complexity represents a priority for the field. Critically, establishing causal inference requires genetic tools that selectively ablate lactylation without disrupting global lactate metabolism or acetylation dynamics. The recent identification of AARS1 and AARS2 as global lysine lactyltransferases offers a potential genetic entry point (21), though whether these enzymes operate in all cell types relevant to sepsis-associated ARDS requires systematic investigation.

The cellular heterogeneity of sepsis responses necessitates single-cell resolution approaches. Current bulk transcriptomic analyses of lactylation-related genes have identified molecular signatures that discriminate septic patients from controls (80, 101), but these studies obscure cell-type-specific contributions. Single-cell lactylation mapping, combining single-cell RNA sequencing with emerging technologies for profiling post-translational modifications at single-cell resolution, would enable resolution of whether distinct immune lineages exhibit characteristic lactylation patterns that drive their functional polarization during sepsis. Complementing this approach, spatial epigenomics could reveal how lactylation marks are distributed across tissue microenvironments. For instance, whether alveolar epithelial cells and adjacent endothelial cells display coordinated or divergent lactylation responses during lung injury remains unexplored. The application of spatial transcriptomics to septic lung tissue could identify lactylation-dependent gene expression programs localized to specific anatomical niches. Moreover, integrating single-cell lactylation profiles with clinical outcomes may refine the lactylation-based phenotypic stratification recently proposed by Wang et al. (119), enabling precision targeting of high-lactylation phenotypes most likely to benefit from intervention.

Preclinical evidence demonstrates that lactylation is pharmacologically modifiable, with studies showing that curcumin ameliorates sepsis-associated acute kidney injury via p300 regulation (113), that gastrodin targets the CDT2-KAT2A axis to treat septic myocardial dysfunction (111), and that exercise-induced histone lactylation restores cardiac immune homeostasis (112). However, several challenges impede clinical translation. The optimal therapeutic window remains undefined, as Yang et al. (22) demonstrated that H3K18 lactylation promotes autophagic gene expression to mitigate immunosuppression in sepsis, indicating that complete ablation of lactylation during the immunoparalysis phase may be harmful. Conversely, excessive lactylation during the hyperinflammatory phase drives organ injury through ferroptosis and NETosis pathways (78, 126). These opposing roles suggest that therapeutic strategies must consider timing and selectivity rather than simply ablating lactylation globally. Additionally, most preclinical studies have evaluated interventions at the time of sepsis induction, whereas patients typically present later in the disease course. Testing therapeutic windows in clinically relevant delayed-intervention models is essential before clinical translation. The specificity of currently available inhibitors for lactylation versus acetylation is also poorly characterized, and the development of substrate-selective inhibitors or PROTAC-mediated degradation strategies targeting specific writers or erasers, as recently reported for Class I HDACs in septic cardiomyopathy (136), offers promise for achieving functional selectivity.

The convergence of multi-omics profiling and machine learning has enabled the identification of lactylation-related hub genes and molecular phenotypes in sepsis-associated ARDS (119, 120). Suo et al. (120) demonstrated that ALDH1A1, CALM1, CCNA2, HIST1H2BN, and SH3GL1 collectively discriminate ARDS patients from septic controls with high diagnostic accuracy. Extending these findings, future studies should integrate lactylation proteomics with metabolomics, transcriptomics, and clinical variables to develop predictive algorithms for patient stratification. The prospective validation of such algorithms in multicenter cohorts represents a necessary step before clinical implementation. Whether lactylation-based molecular phenotypes predict differential responses to targeted therapies remains unknown, and answering this question will require embedding biomarker analyses within future interventional trials.

In summary, the future of lactylation research in sepsis-associated ARDS lies in moving from correlation to causation, from bulk to single-cell resolution, and from descriptive phenotyping to mechanism-guided therapeutic targeting. Addressing these challenges will require interdisciplinary collaboration among epigeneticists, immunologists, bioinformaticians, and clinician-scientists. The integration of emerging technologies including single-cell epigenomics, spatial transcriptomics, and machine learning with rigorous preclinical and clinical validation holds promise for translating the lactylation bridge into actionable strategies that improve outcomes for patients with this devastating syndrome.

## Glossary

AARS1: alanyl-tRNA synthetase 1
AARS2: alanyl-tRNA synthetase 2
ACSS2: acyl-CoA synthetase short-chain family member 2
ALDH1A1: aldehyde dehydrogenase 1 family member A1
ARDS: acute respiratory distress syndrome
BCAP: B-cell adapter for PI3K
CALM1: calmodulin 1
CCNA2: cyclin A2
CCT2: chaperonin containing TCP1 subunit 2
cGAS: cyclic GMP-AMP synthase
CIRP: cold-inducible RNA-binding protein
eEF1A2: eukaryotic translation elongation factor 1 alpha 2
FGF2: fibroblast growth factor 2
FGF15: fibroblast growth factor 15
FGFR4: fibroblast growth factor receptor 4
GPD2: glycerol−3−phosphate dehydrogenase 2
GTPSCS: GTP−specific succinyl−CoA synthetase
H3K9la: histone H3 lysine 9 lactylation
H3K14la: histone H3 lysine 14 lactylation
H3K18la: histone H3 lysine 18 lactylation
HAT1: histone acetyltransferase 1
HBO1: histone acetyltransferase binding to ORC1
HDAC: histone deacetylase
HIST1H2BN: histone cluster 1 H2B family member N
HMGB1: high mobility group box 1
KAT2A: lysine acetyltransferase 2A
KAT8: lysine acetyltransferase 8
LDHB: lactate dehydrogenase B
LDHA: lactate dehydrogenase A
LPCAT2: lysophosphatidylcholine acyltransferase 2
LRG: lactylation−related gene
MCT4: monocarboxylate transporter 4
MDSC: myeloid−derived suppressor cell
METTL3: methyltransferase−like 3
NAD: nicotinamide adenine dinucleotide
NET: neutrophil extracellular trap
NETosis: neutrophil extracellular trap formation
NLRP3: NLR family pyrin domain containing 3
NR4A3: nuclear receptor subfamily 4 group A member 3
PAD4: peptidyl arginine deiminase 4
PDK4: pyruvate dehydrogenase kinase 4
PROTAC: proteolysis targeting chimera
PSMD14: proteasome 26S subunit, non−ATPase 14
RBM25: RNA−binding motif protein 25
RORα: RAR−related orphan receptor alpha
RPA1: replication protein A1
RUBCNL: rubicon like autophagy enhancer
SH3GL1: SH3 domain containing GRB2 like 1
SIRT1: sirtuin 1
SIRT2: sirtuin 2
SLC40A1: solute carrier family 40 member 1 (ferroportin)
TLR: Toll−like receptor
TRIM33: tripartite motif−containing 33
WTAP: Wilms tumor 1 associated protein
YY1: ZBP1, Z−DNA binding protein 1

## References

[1] Wang Z Wang Z . The role of macrophages polarization in sepsis-induced acute lung injury. Front Immunol. (2023) 14:1209438. doi: 10.3389/fimmu.2023.1209438 37691951 PMC10483837

[2] Tang J Yan M Li M Hu Z Zhou K . From "metabolic storm" to "immune paralysis": the dynamic evolution of macrophages and metabolism reprogramming in ARDS. Front Immunol. (2025) 16:1738713. doi: 10.3389/fimmu.2025.1738713 41488672 PMC12758027

[3] Cheng S Li Y Sun X Liu Z Guo L Wu J . The impact of glucose metabolism on inflammatory processes in sepsis-induced acute lung injury. Front Immunol. (2024) 15:1508985. doi: 10.3389/fimmu.2024.1508985 39712019 PMC11659153

[4] Zhang S Hu B Fang Y Liu M Liu Q Chen Y . Decoding the HIF-1-driven metabolic-inflammatory-immune axis in sepsis-associated lung injury: a comprehensive overview. Front Immunol. (2025) 16:1658103. doi: 10.3389/fimmu.2025.1658103 41562069 PMC12812678

[5] Sun L Zhang H Gao P . Metabolic reprogramming and epigenetic modifications on the path to cancer. Protein Cell. (2022) 13:877–919. doi: 10.1007/s13238-021-00846-7 34050894 PMC9243210

[6] Xu X Peng Q Jiang X Tan S Yang Y Yang W . Metabolic reprogramming and epigenetic modifications in cancer: from the impacts and mechanisms to the treatment potential. Exp Mol Med. (2023) 55:1357–70. doi: 10.1038/s12276-023-01020-1 37394582 PMC10394076

[7] Wu D Zhang K Khan FA Wu Q Pandupuspitasari NS Tang Y . The emerging era of lactate: a rising star in cellular signaling and its regulatory mechanisms. J Cell Biochem. (2023) 124:1067–81. doi: 10.1002/jcb.30458 37566665

[8] Zhang T Chen L Kueth G Shao E Wang X Ha T . Lactate's impact on immune cells in sepsis: unraveling the complex interplay. Front Immunol. (2024) 15:1483400. doi: 10.3389/fimmu.2024.1483400 39372401 PMC11449721

[9] Wang T Ye Z Li Z Jing DS Fan GX Liu MQ . Lactate-induced protein lactylation: a bridge between epigenetics and metabolic reprogramming in cancer. Cell Prolif. (2023) 56:e13478. doi: 10.1111/cpr.13478 37060186 PMC10542650

[10] Zhang D Tang Z Huang H Zhou G Cui C Weng Y . Metabolic regulation of gene expression by histone lactylation. Nature. (2019) 574:575–80. doi: 10.1038/s41586-019-1678-1 31645732 PMC6818755

[11] Xin Q Wang H Li Q Liu S Qu K Liu C . Lactylation: a passing fad or the future of posttranslational modification. Inflammation. (2022) 45:1419–29. doi: 10.1007/s10753-022-01637-w 35224683 PMC9197907

[12] Irizarry-Caro RA McDaniel MM Overcast GR Jain VG Troutman TD Pasare C . TLR signaling adapter BCAP regulates inflammatory to reparatory macrophage transition by promoting histone lactylation. Proc Natl Acad Sci USA. (2020) 117:30628–38. doi: 10.1073/pnas.2009778117 33199625 PMC7720107

[13] Chu X Di C Chang P Li L Feng Z Xiao S . Lactylated histone H3K18 as a potential biomarker for the diagnosis and predicting the severity of septic shock. Front Immunol. (2021) 12:786666. doi: 10.3389/fimmu.2021.786666 35069560 PMC8773995

[14] Lv M Huang Y Chen Y Ding K . Lactylation modification in cancer: mechanisms, functions, and therapeutic strategies. Exp Hematol Oncol. (2025) 14:32. doi: 10.1186/s40164-025-00622-x 40057816 PMC11889934

[15] Liu Z Li A Ma Z Wang J Chen X Wang Z . Lactate metabolism and protein lactylation in cancer. Mol BioMed. (2026) 7:15. doi: 10.1186/s43556-026-00417-4 41746580 PMC12946641

[16] Rong Y Dong F Zhang G Tang M Zhao X Zhang Y . The crosstalking of lactate-histone lactylation and tumor. Proteomics Clin Appl. (2023) 17:e2200102. doi: 10.1002/prca.202200102 36853081

[17] Dai E Wang W Li Y Ye D Li Y . Lactate and lactylation: behind the development of tumors. Cancer Lett. (2024) 591:216896. doi: 10.1016/j.canlet.2024.216896 38641309

[18] Zhao L Qi H Lv H Liu W Zhang R Yang A . Lactylation in health and disease: physiological or pathological? Theranostics. (2025) 15:1787–821. doi: 10.7150/thno.105353 39897556 PMC11780532

[19] Zong Z Zhang L Zhou F . Lactylation in cancer: advances, challenges, and future perspectives. Cancer Res. (2025) 85:3192–5. doi: 10.1158/0008-5472.Can-24-4394 40891207

[20] Zhang C Meng Q Jiao H Liu H Wang X Zhou H . Lactylation in cancer: metabolic-epigenetic nexus and therapeutic frontiers. Crit Rev Oncol Hematol. (2026) 217:105034. doi: 10.1016/j.critrevonc.2025.105034 41270849

[21] Fang Z Zhu GS Nie DY Xu B . Lactylation modification - a bridge between sepsis and macrophage metabolic reprogramming. Front Immunol. (2026) 17:1738765. doi: 10.3389/fimmu.2026.1738765 41869341 PMC13002459

[22] Yang T Liu S Jiang Q Wang N Zhang L Shi X . Lactylation of histone H3K18 promotes autophagic gene expression to mitigate immunosuppression in sepsis. Int J Biol Macromol. (2026) 358:151667. doi: 10.1016/j.ijbiomac.2026.151667 41895498

[23] Lu Z Fang P Li S Xia D Zhang J Wu X . Lactylation of histone H3k18 and Egr1 promotes endothelial glycocalyx degradation in sepsis-induced acute lung injury. Adv Sci (Weinh). (2025) 12:e2407064. doi: 10.1002/advs.202407064 39721014 PMC11831459

[24] Yan K Mousavi N Yang XJ . Analysis of lysine acetylation and acetylation-like acylation *in vitro* and *in vivo*. Curr Protoc. (2023) 3:e738. doi: 10.1002/cpz1.738 37184117

[25] Sheng X Lin H Cole PA Zhao Y . Biochemistry and regulation of histone lysine L-lactylation. Nat Rev Mol Cell Biol. (2026) 27:95–109. doi: 10.1038/s41580-025-00876-7 40830268 PMC12920031

[26] Zhang D Gao J Zhu Z Mao Q Xu Z Singh PK . Lysine L-lactylation is the dominant lactylation isomer induced by glycolysis. Nat Chem Biol. (2025) 21:91–9. doi: 10.1038/s41589-024-01680-8 39030363 PMC11666458

[27] Moreno-Yruela C Bæk M Monda F Olsen CA . Chiral posttranslational modification to lysine ϵ-amino groups. Acc Chem Res. (2022) 55:1456–66. doi: 10.1021/acs.accounts.2c00115 35500056

[28] Liu R Ren X Park YE Feng H Sheng X Song X . Nuclear GTPSCS functions as a lactyl-CoA synthetase to promote histone lactylation and gliomagenesis. Cell Metab. (2025) 37:377–394.e9. doi: 10.1016/j.cmet.2024.11.005 39642882 PMC11798710

[29] Zhu R Ye X Lu X Xiao L Yuan M Zhao H . ACSS2 acts as a lactyl-CoA synthetase and couples KAT2A to function as a lactyltransferase for histone lactylation and tumor immune evasion. Cell Metab. (2025) 37:361–376.e7. doi: 10.1016/j.cmet.2024.10.015 39561764

[30] Li H Liu C Li R Zhou L Ran Y Yang Q . AARS1 and AARS2 sense L-lactate to regulate cGAS as global lysine lactyltransferases. Nature. (2024) 634:1229–37. doi: 10.1038/s41586-024-07992-y 39322678

[31] Zong Z Xie F Wang S Wu X Zhang Z Yang B . Alanyl-tRNA synthetase, AARS1, is a lactate sensor and lactyltransferase that lactylates p53 and contributes to tumorigenesis. Cell. (2024) 187:2375–2392.e33. doi: 10.1016/j.cell.2024.04.002 38653238

[32] Zong Z Ren J Yang B Zhang L Zhou F . Emerging roles of lysine lactyltransferases and lactylation. Nat Cell Biol. (2025) 27:563–74. doi: 10.1038/s41556-025-01635-8 40185947

[33] Niu Z Chen C Wang S Lu C Wu Z Wang A . HBO1 catalyzes lysine lactylation and mediates histone H3K9la to regulate gene transcription. Nat Commun. (2024) 15:3561. doi: 10.1038/s41467-024-47900-6 38670996 PMC11053077

[34] Xie B Zhang M Li J Cui J Zhang P Liu F . KAT8-catalyzed lactylation promotes eEF1A2-mediated protein synthesis and colorectal carcinogenesis. Proc Natl Acad Sci USA. (2024) 121:e2314128121. doi: 10.1073/pnas.2314128121 38359291 PMC10895275

[35] He J Lai T Zhao Y Zhou Z Zhou L Tao D . HAT1 functions as a lactyltransferase and mediates RPA1 lactylation to promote DNA repair and radioresistance in lung adenocarcinoma. Cell Death Dis. (2025) 16:851. doi: 10.1038/s41419-025-08113-x 41271679 PMC12639135

[36] Moreno-Yruela C Zhang D Wei W Bæk M Liu W Gao J . Class I histone deacetylases (HDAC1-3) are histone lysine delactylases. Sci Adv. (2022) 8:eabi6696. doi: 10.1126/sciadv.abi6696 35044827 PMC8769552

[37] Du R Gao Y Yan C Ren X Qi S Liu G . Sirtuin 1/sirtuin 3 are robust lysine delactylases and sirtuin 1-mediated delactylation regulates glycolysis. iScience. (2024) 27:110911. doi: 10.1016/j.isci.2024.110911 39351192 PMC11440250

[38] Fan Z Liu Z Zhang N Wei W Cheng K Sun H . Identification of SIRT3 as an eraser of H4K16la. iScience. (2023) 26:107757. doi: 10.1016/j.isci.2023.107757 37720100 PMC10504495

[39] Chen C Zhang Y Zang Y Fan Z Han Y Bai X . SIRT3 functions as an eraser of histone H3K9 lactylation to modulate transcription for inhibiting the progression of esophageal cancer. Mol Cell Proteomics. (2025) 24:100973. doi: 10.1016/j.mcpro.2025.100973 40252727 PMC12144510

[40] Nickel GA Pederson NJ Faheem Yang Z Bulf J Diehl KL . Sirtuin 6 is a histone delactylase. J Biol Chem. (2025) 301:110795. doi: 10.1016/j.jbc.2025.110795 41062064 PMC12664031

[41] Tsusaka T Najar MA Sharma I Marcinkiewicz MM Crispim C Snyder NW . Class I histone deacetylases catalyze lysine lactylation. bioRxiv. (2025) 28. doi: 10.1101/2025.02.25.640220 40835008 PMC12624779

[42] Zhai G Niu Z Jiang Z Zhao F Wang S Chen C . DPF2 reads histone lactylation to drive transcription and tumorigenesis. Proc Natl Acad Sci USA. (2024) 121:e2421496121. doi: 10.1073/pnas.2421496121 39636855 PMC11648877

[43] Nuñez R Sidlowski PFW Steen EA Wynia-Smith SL Sprague DJ Keyes RF . The TRIM33 bromodomain recognizes histone lysine lactylation. ACS Chem Biol. (2024) 19:2418–28. doi: 10.1021/acschembio.4c00248 39556662 PMC11706526

[44] Hu X Huang X Yang Y Sun Y Zhao Y Zhang Z . Dux activates metabolism-lactylation-MET network during early iPSC reprogramming with Brg1 as the histone lactylation reader. Nucleic Acids Res. (2024) 52:5529–48. doi: 10.1093/nar/gkae183 38512058 PMC11162783

[45] Zhao Y Zhang M Huang X Liu J Sun Y Zhang F . Lactate modulates zygotic genome activation through H3K18 lactylation rather than H3K27 acetylation. Cell Mol Life Sci. (2024) 81:298. doi: 10.1007/s00018-024-05349-2 38992327 PMC11335220

[46] Di C Chu X Chang P Zhao Y Chong J Chen S . The roles of histone H3K18 lactylation, acetylation, and lactylation/acetylation ratio as potential biomarkers in the diagnosis and severity assessment of sepsis and septic shock. Infect Dis Ther. (2025) 14:2785–818. doi: 10.1007/s40121-025-01232-0 41085943 PMC12602787

[47] Cross D Drury R Hill J Pollard AJ . Epigenetics in sepsis: understanding its role in endothelial dysfunction, immunosuppression, and potential therapeutics. Front Immunol. (2019) 10:1363. doi: 10.3389/fimmu.2019.01363 31275313 PMC6591469

[48] Wu D Shi Y Zhang H Miao C . Epigenetic mechanisms of immune remodeling in sepsis: targeting histone modification. Cell Death Dis. (2023) 14:112. doi: 10.1038/s41419-023-05656-9 36774341 PMC9922301

[49] Visan I . Histone lactylation. Nat Immunol. (2019) 20:1558. doi: 10.1038/s41590-019-0551-6 31745344

[50] Izzo LT Wellen KE . Histone lactylation links metabolism and gene regulation. Nature. (2019) 574:492–3. doi: 10.1038/d41586-019-03122-1 31645737

[51] Rho H Terry AR Chronis C Hay N . Hexokinase 2-mediated gene expression via histone lactylation is required for hepatic stellate cell activation and liver fibrosis. Cell Metab. (2023) 35:1406–1423.e8. doi: 10.1016/j.cmet.2023.06.013 37463576 PMC11748916

[52] Li F Si W Xia L Yin D Wei T Tao M . Positive feedback regulation between glycolysis and histone lactylation drives oncogenesis in pancreatic ductal adenocarcinoma. Mol Cancer. (2024) 23:90. doi: 10.1186/s12943-024-02008-9 38711083 PMC11071201

[53] Jiang J Huang D Jiang Y Hou J Tian M Li J . Lactate modulates cellular metabolism through histone lactylation-mediated gene expression in non-small cell lung cancer. Front Oncol. (2021) 11:647559. doi: 10.3389/fonc.2021.647559 34150616 PMC8208031

[54] Li X Yang N Wu Y Wang X Sun J Liu L . Hypoxia regulates fibrosis-related genes via histone lactylation in the placentas of patients with preeclampsia. J Hypertens. (2022) 40:1189–98. doi: 10.1097/hjh.0000000000003129 35703881

[55] De Leo A Ugolini A Yu X Scirocchi F Scocozza D Peixoto B . Glucose-driven histone lactylation promotes the immunosuppressive activity of monocyte-derived macrophages in glioblastoma. Immunity. (2024) 57:1105–1123.e8. doi: 10.1016/j.immuni.2024.04.006 38703775 PMC11114377

[56] Desgeorges T Galle E Zhang J von Meyenn F De Bock K . Histone lactylation in macrophages is predictive for gene expression changes during ischemia induced-muscle regeneration. Mol Metab. (2024) 83:101923. doi: 10.1016/j.molmet.2024.101923 38521183 PMC11002880

[57] Fan M Yang K Wang X Chen L Gill PS Ha T . Lactate promotes endothelial-to-mesenchymal transition via Snail1 lactylation after myocardial infarction. Sci Adv. (2023) 9:eadc9465. doi: 10.1126/sciadv.adc9465 36735787 PMC9897666

[58] Yu T Li X Wang C Yang Y Fu X Li T . Lactylation of mitochondrial adenosine triphosphate synthase subunit alpha regulates vascular remodeling and progression of aortic dissection. Res (Wash D C). (2025) 8:799. doi: 10.34133/research.0799 40800583 PMC12342782

[59] Zhang D Liang C Wu C Hawanga M Wan S Xu L . Nonhistone lactylation: A hub for tumour metabolic reprogramming and epigenetic regulation. J Transl Med. (2025) 23:901. doi: 10.1186/s12967-025-06813-8 40797262 PMC12344884

[60] Peng X Du J . Histone and non-histone lactylation: molecular mechanisms, biological functions, diseases, and therapeutic targets. Mol BioMed. (2025) 6:38. doi: 10.1186/s43556-025-00275-6 40484921 PMC12146230

[61] Wu D Spencer CB Ortoga L Zhang H Miao C . Histone lactylation-regulated METTL3 promotes ferroptosis via m6A-modification on ACSL4 in sepsis-associated lung injury. Redox Biol. (2024) 74:103194. doi: 10.1016/j.redox.2024.103194 38852200 PMC11219935

[62] Li W Zhou C Yu L Hou Z Liu H Kong L . Tumor-derived lactate promotes resistance to bevacizumab treatment by facilitating autophagy enhancer protein RUBCNL expression through histone H3 lysine 18 lactylation (H3K18la) in colorectal cancer. Autophagy. (2024) 20:114–30. doi: 10.1080/15548627.2023.2249762 37615625 PMC10761097

[63] Xu L Ye Y Gu W Xu X Chen N Zhang L . Histone lactylation stimulated upregulation of PSMD14 alleviates neuron PANoptosis through deubiquitinating PKM2 to activate PINK1-mediated mitophagy after traumatic brain injury. Autophagy. (2025) 21:1473–91. doi: 10.1080/15548627.2025.2471633 40000916 PMC12283019

[64] Fan W Zeng S Wang X Wang G Liao D Li R . A feedback loop driven by H3K9 lactylation and HDAC2 in endothelial cells regulates VEGF-induced angiogenesis. Genome Biol. (2024) 25:165. doi: 10.1186/s13059-024-03308-5 38918851 PMC11197246

[65] Wang X Fan W Li N Ma Y Yao M Wang G . YY1 lactylation in microglia promotes angiogenesis through transcription activation-mediated upregulation of FGF2. Genome Biol. (2023) 24:87. doi: 10.1186/s13059-023-02931-y 37085894 PMC10120156

[66] Ma W Jia K Cheng H Xu H Li Z Zhang H . Orphan nuclear receptor NR4A3 promotes vascular calcification via histone lactylation. Circ Res. (2024) 134:1427–47. doi: 10.1161/circresaha.123.323699 38629274

[67] Zang G Xu S Sun Z Zhang L Qian Y Yao H . IDH2 lactylation promotes angiogenesis in murine diabetic myocardial infarction via blocking Cav1-eNOS interaction. Nat Commun. (2025) 17:1117. doi: 10.1038/s41467-025-67877-0 41419771 PMC12855196

[68] Wu J Hu M Jiang H Ma J Xie C Zhang Z . Endothelial cell-derived lactate triggers bone mesenchymal stem cell histone lactylation to attenuate osteoporosis. Adv Sci (Weinh). (2023) 10:e2301300. doi: 10.1002/advs.202301300 37752768 PMC10625121

[69] Yang YH Wang QC Kong J Yang JT Liu JF . Global profiling of lysine lactylation in human lungs. Proteomics. (2023) 23:e2200437. doi: 10.1002/pmic.202200437 37170646

[70] Zhang Y Jiang H Dong M Min J He X Tan Y . Macrophage MCT4 inhibition activates reparative genes and protects from atherosclerosis by histone H3 lysine 18 lactylation. Cell Rep. (2024) 43:114180. doi: 10.1016/j.celrep.2024.114180 38733581

[71] Li B Li J Zhu Z Tang Y Zhou Y Du G . FGF15/FGFR4 signaling suppresses M1 macrophage polarization and multi-organ inflammation in septic mice by inhibiting H3K18 lactylation-driven Irf7 expression through NF2-Hippo activation. Cell Death Dis. (2025) 16:628. doi: 10.1038/s41419-025-07962-w 40825908 PMC12361455

[72] Chen L Zhang M Yang X Wang Y Huang T Li X . Methyl-CpG-binding 2 K271 lactylation-mediated M2 macrophage polarization inhibits atherosclerosis. Theranostics. (2024) 14:4256–77. doi: 10.7150/thno.94738 39113793 PMC11303070

[73] Huang C Xue L Lin X Shen Y Wang X . Histone lactylation-driven GPD2 mediates M2 macrophage polarization to promote Malignant transformation of cervical cancer progression. DNA Cell Biol. (2024) 43:605–18. doi: 10.1089/dna.2024.0122 39504115

[74] Guo H Luan N Gao J Pang X Bi J Zhu L . Exploring the mechanisms of mutual influence between lactylation and macrophage polarization in the context of disease. Clin Transl Med. (2025) 15:e70499. doi: 10.1002/ctm2.70499 41190507 PMC12587168

[75] Shu M Lu D Zhu Z Yang F Ma Z . Insight into the roles of lactylation in macrophages: functions and clinical implications. Clin Sci (Lond). (2025) 139:151–69. doi: 10.1042/cs20242737 39876839 PMC12204002

[76] Qiu CZ Zhou R Zhang HY Zhang L Yin ZJ Ren DL . Histone lactylation-ROS loop contributes to light exposure-exacerbated neutrophil recruitment in zebrafish. Commun Biol. (2024) 7:887. doi: 10.1038/s42003-024-06543-5 39033200 PMC11271584

[77] Zhu L Zheng Q Liu X Ding H Ma M Bao J . HMGB1 lactylation drives neutrophil extracellular trap formation in lactate-induced acute kidney injury. Front Immunol. (2024) 15:1475543. doi: 10.3389/fimmu.2024.1475543 39850900 PMC11754054

[78] Huang WL Wang YC Cheng TL Tang C Liu ZW Xu Y . Histone lactylation-mediated PAD4 up-regulation promotes septic acute kidney injury via activating NETosis. Nephrol (Carlton). (2025) 30:e70137. doi: 10.1111/nep.70137 41103221

[79] Wei S Dai Z Wu L Xiang Z Yang X Jiang L . Lactate-induced macrophage HMGB1 lactylation promotes neutrophil extracellular trap formation in sepsis-associated acute kidney injury. Cell Biol Toxicol. (2025) 41:78. doi: 10.1007/s10565-025-10026-6 40304798 PMC12043764

[80] Wang H Yang R Chen N Li X . Heterogeneity of neutrophils and immunological function in neonatal sepsis: Analysis of molecular subtypes based on hypoxia-glycolysis-lactylation. Mediators Inflammation. (2025) 2025:5790261. doi: 10.1155/mi/5790261 40177399 PMC11964727

[81] Zhou R Ding RC Yu Q Qiu CZ Zhang HY Yin ZJ . Metformin attenuates neutrophil recruitment through the H3K18 lactylation/reactive oxygen species pathway in zebrafish. Antiox (Basel). (2024) 13:176. doi: 10.3390/antiox13020176 38397774 PMC10886385

[82] Zhou R Li K Hu X Fan S Gao Y Xue X . Sleep deprivation activates a conserved lactate-H3K18la-RORα axis driving neutrophilic inflammation across species. Adv Sci (Weinh). (2025) 12:e04028. doi: 10.1002/advs.202504028 40686333 PMC12520482

[83] Da W Dai Y Shen B Zhang Y Bao P Zhu W . Histone lactylation-derived TET2 enhanced Arg1-mediated MDSC immunosuppression. Front Immunol. (2025) 16:1677780. doi: 10.3389/fimmu.2025.1677780 41624845 PMC12855476

[84] Zhang X Wang WB Cai XY Chen XQ Feng Q Yang Q . MNDA promotes immunosuppression in microsatellite instability-high colorectal cancer by facilitating PMN-MDSC infiltration via H3K18 lactylation. J Transl Med. (2025) 23:1049. doi: 10.1186/s12967-025-07097-8 41044625 PMC12495719

[85] Babl N Voll F Martowicz A Bruss C Maddaloni M Schmidl C . Lactic acid promotes an MDSC-like phenotype via HIF1α stabilization with impact on prognosis in renal cell carcinoma. Cancer Lett. (2026) 639:218209. doi: 10.1016/j.canlet.2025.218209 41371433

[86] Liu X Chen Y Liu W Pan J Liu J Dong X . High throughput screening identifies sanguinarine chloride as a multi-faceted therapeutic agent for PRCC-TFE3 rRCC by targeting lactylation-driven VEGFB-VEGFR2 signaling and PMN-MDSC infiltration. J Adv Res. (2026) 26:00291–2. doi: 10.1016/j.jare.2026.04.012 41956242

[87] Bar-Or D Banton K Acuna D Williams J Palacio CH Zaw-Mon C . Lactylation as a metabolic-epigenetic switch: Mechanisms and roles in cancer, sepsis, trauma, inflammation, and tissue repair. Biochem Biophys Rep. (2026) 45:102507. doi: 10.1016/j.bbrep.2026.102507 41732414 PMC12925563

[88] Fan W Wang X Zeng S Li N Wang G Li R . Global lactylome reveals lactylation-dependent mechanisms underlying T(H)17 differentiation in experimental autoimmune uveitis. Sci Adv. (2023) 9:adh4655. doi: 10.1126/sciadv.adh4655 37851814 PMC10584346

[89] Lopez Krol A Nehring HP Krause FF Wempe A Raifer H Nist A . Lactate induces metabolic and epigenetic reprogramming of pro-inflammatory Th17 cells. EMBO Rep. (2022) 23:e54685. doi: 10.15252/embr.202254685 36215678 PMC9724659

[90] Raychaudhuri D Singh P Chakraborty B Hennessey M Tannir AJ Byregowda S . Histone lactylation drives CD8(+) T cell metabolism and function. Nat Immunol. (2024) 25:2140–51. doi: 10.1038/s41590-024-01985-9 39375549 PMC13211864

[91] Wang R Li C Cheng Z Li M Shi J Zhang Z . H3K9 lactylation in Malignant cells facilitates CD8(+) T cell dysfunction and poor immunotherapy response. Cell Rep. (2024) 43:114686. doi: 10.1016/j.celrep.2024.114686 39216002

[92] Sun R Chi H . Metabolic-epigenetic rewiring in CD8(+) T cells via lactate-dependent histone lactylation. Nat Immunol. (2024) 25:1980–2. doi: 10.1038/s41590-024-01991-x 39415052

[93] Pignataro G Triunfo C Piccioni A Racco S Fuorlo M Forte E . The epigenetics of sepsis: How gene modulation shapes outcomes. Biomedicines. (2025) 13:1936. doi: 10.3390/biomedicines13081936 40868190 PMC12383537

[94] Hao ZN Tan XP Zhang Q Li J Xia R Ma Z . Lactate and lactylation: Dual regulators of T-cell-mediated tumor immunity and immunotherapy. Biomolecules. (2024) 14:1646. doi: 10.3390/biom14121646 39766353 PMC11674224

[95] Xie L Deng X Li X Li X Wang X Yan H . CircMETTL3-156aa reshapes the glycolytic metabolism of macrophages to promote M1 polarization and induce cytokine storms in sHLH. Cell Death Discov. (2024) 10:431. doi: 10.1038/s41420-024-02202-0 39384750 PMC11464708

[96] Susser LI Nguyen MA Geoffrion M Emerton C Ouimet M Khacho M . Mitochondrial fragmentation promotes inflammation resolution responses in macrophages via histone lactylation. Mol Cell Biol. (2023) 43:531–46. doi: 10.1080/10985549.2023.2253131 37807652 PMC10569354

[97] Jiang J Wang R Song P Peng Q Jin X Li B . Lactate facilitates pancreatic repair following acute pancreatitis by promoting reparative macrophage polarization. Cell Mol Gastroenterol Hepatol. (2025) 19:101535. doi: 10.1016/j.jcmgh.2025.101535 40350150 PMC12213952

[98] Sun S Xu X Liang L Wang X Bai X Zhu L . Lactic acid-producing probiotic Saccharomyces cerevisiae attenuates ulcerative colitis via suppressing macrophage pyroptosis and modulating gut microbiota. Front Immunol. (2021) 12:777665. doi: 10.3389/fimmu.2021.777665 34899735 PMC8652295

[99] Guo Y Chu L Shui W Hu H Hao L Wang D . Histone lactylation in immune cells and its predictive role in sepsis progression: A prospective observational study. Shock. (2025) 65:792–800. doi: 10.1097/shk.0000000000002659 40663442 PMC13132054

[100] Yang Y Zhang Y Liu K Zhang H Zuo X Guo M . Lactate and lactylation in immune cell function and autoimmune diseases: Mechanisms and therapeutic potential. Immunology. (2026) 177:643–61. doi: 10.1111/imm.70075 41401978

[101] Li Y Zhang T Zhou L Huang Y Li J Xu H . Identification and prognostic potential of lactylation-related genes in sepsis: implications of the RBM25-acly axis. Int Immunopharmacol. (2025) 162:115177. doi: 10.1016/j.intimp.2025.115177 40651437

[102] Li X Shang Y Zhang J Mu G Duan Y Lu Z . Predictive value of H3K18 lactylation for early detection and prognosis of sepsis-related acute respiratory distress syndrome: a prospective observational clinical study. Shock. (2025) 64:154–60. doi: 10.1097/shk.0000000000002601 40267500

[103] Li S Shen Y Wang C Yang J Chen M Hu Y . Exploring the prognostic and diagnostic value of lactylation-related genes in sepsis. Sci Rep. (2024) 14:23130. doi: 10.1038/s41598-024-74040-0 39367086 PMC11452377

[104] Jiang K Mai S Li J Zhou H Chen Y Zou L . Exploring the molecular mechanisms of lactylation-related biological functions and immune regulation in sepsis-associated acute kidney injury. Clin Exp Med. (2025) 25:200. doi: 10.1007/s10238-025-01745-5 40504273 PMC12162736

[105] Jin R Dai J Zhang X Chen Y Xiong W Qian Z . Integrative analysis identifies lactylation-associated hub genes in septic cardiomyopathy. Shock. (2026). doi: 10.1097/shk.0000000000002819 41886545

[106] An S Yao Y Hu H Wu J Li J Li L . PDHA1 hyperacetylation-mediated lactate overproduction promotes sepsis-induced acute kidney injury via Fis1 lactylation. Cell Death Dis. (2023) 14:457. doi: 10.1038/s41419-023-05952-4 37479690 PMC10362039

[107] Huang Y Zhao E Zhao G Zhuo W Zhao Y Wang H . H3K18 lactylation-mediated SPHK1-SIRT1 feedback loop accelerates pyroptosis of tubular epithelial cells in sepsis-associated acute kidney injury. Theranostics. (2026) 16:4768–86. doi: 10.7150/thno.122991 41799201 PMC12964219

[108] Qiao J Tan Y Liu H Yang B Zhang Q Liu Q . Histone H3K18 and Ezrin lactylation promote renal dysfunction in sepsis-associated acute kidney injury. Adv Sci (Weinh). (2024) 11:e2307216. doi: 10.1002/advs.202307216 38767134 PMC11267308

[109] Luo M Mi Z Yu B Zhang S Wu P Shi Y . LDHB K156 lactylation links cGAS-STING-mediated metabolic reprogramming to NLRP3 inflammasome activation in sepsis-associated acute kidney injury. Life Sci. (2026) 392:124311. doi: 10.1016/j.lfs.2026.124311 41796892

[110] Zhong Y Shen S You Q Wang J Wu Q Zhang L . Mitochondrial regulation of lactylation in sepsis-induced cardiomyopathy. Crit Care. (2025) 30:31. doi: 10.1186/s13054-025-05802-z 41408319 PMC12821899

[111] Yu H Du Q Wu J Feng F Hou S Liu M . Gastrodin regulates H3K14la through the CDT2-KAT2A axis to treat sepsis-induced myocardial dysfunction. Int Immunopharmacol. (2025) 161:115065. doi: 10.1016/j.intimp.2025.115065 40532326

[112] Sun S Lai C Huang C Ren X Zhang T Zou J . Exercise-induced histone lactylation in monocyte-derived macrophages restores cardiac immune homeostasis and function in sepsis-induced cardiomyopathy. Nat Commun. (2025) 17:756. doi: 10.1038/s41467-025-67443-8 41398160 PMC12819526

[113] Luo M Zhu Q Xu G Liu D Xiao J Shi Q . Intervention effect of curcumin on sepsis-associated acute kidney injury via regulation of p300 expression and protein lactylation. BMC Immunol. (2025) 26:67. doi: 10.1186/s12865-025-00750-3 40993513 PMC12459039

[114] Mokhtari B Alihemmati A Bafadam S Boraghi S Badalzadeh R Mahmoodpoor A . Mitotherapy attenuates sepsis-induced brain injury in rats subjected to cecal ligation and puncture: Role of SIRT-1/PGC-1α network. Iran J Bas Med Sci. (2025) 28:1065–74. doi: 10.22038/ijbms.2025.84848.18363 40584438 PMC12203824

[115] Luo S Lyu Z Huang H Zhao J Li Y Luo Y . Targeting AARS1-dependent lactylation improves neuronal process plasticity and mitigates cognitive deficits in sepsis-associated encephalopathy. Brain Behav Immun. (2026) 135:106493. doi: 10.1016/j.bbi.2026.106493 41713664

[116] Gong T Wang QD Loughran PA Li YH Scott MJ Billiar TR . Mechanism of lactic acidemia-promoted pulmonary endothelial cells death in sepsis: role for CIRP-ZBP1-PANoptosis pathway. Mil Med Res. (2024) 11:71. doi: 10.1186/s40779-024-00574-z 39465383 PMC11514876

[117] Gong F Zheng X Xu W Xie R Liu W Pei L . H3K14la drives endothelial dysfunction in sepsis-induced ARDS by promoting SLC40A1/transferrin-mediated ferroptosis. MedComm (2020). (2025) 6:e70049. doi: 10.1002/mco2.70049 39822760 PMC11733091

[118] Qin KW Ji QQ Luo WJ Li WQ Hao BB Zheng HY . Sirtuin 3 attenuates acute lung injury by decreasing ferroptosis and inflammation through inhibiting aerobic glycolysis. BioMed Environ Sci. (2025) 38:1161–7. doi: 10.3967/bes2025.110 41088822

[119] Wang Y Wei A Su Z Shi Y Li X He L . Characterization of lactylation-based phenotypes and molecular biomarkers in sepsis-associated acute respiratory distress syndrome. Sci Rep. (2025) 15:13831. doi: 10.1038/s41598-025-96969-6 40263316 PMC12015483

[120] Suo T Xu M Fang J . Lactylation modulates immune infiltration in sepsis-induced acute respiratory distress syndrome: a multi-omics and machine learning study with experimental confirmation. Eur J Med Res. (2025) 30:1100. doi: 10.1186/s40001-025-03355-z 41214769 PMC12604238

[121] Qian H Huang C Meng J Sun Z Nie S Xue X . Integrative analysis identifies lactylation-related biomarkers in sepsis via bioinformatics and machine learning. Biochem Biophys Res Commun. (2026) 801:153296. doi: 10.1016/j.bbrc.2026.153296 41570698

[122] Lin P Wang Y Li X Liang Z Wang T . Histone H3 lysine 18 lactylation promotes alveolar epithelial cell apoptosis in sepsis-induced lung injury by upregulating caspase-8 *in vivo* and *in vitro*. Exp Cell Res. (2026) 454:114843. doi: 10.1016/j.yexcr.2025.114843 41320149

[123] Du B Song W Zhang Y Yin Y Zhou Y Pan Y . Upregulation of m(6)A writer WTAP by histone lactylation promotes inflammatory response via TLR2 in neutrophils. Sci China Life Sci. (2026) 69:1578–93. doi: 10.1007/s11427-024-3081-9 41569386

[124] Zhang J Wu D Zeng F Gu H Li C Cata JP . Lactate metabolic reprogramming and histone lactylation modification in sepsis. Int J Biol Sci. (2025) 21:5034–55. doi: 10.7150/ijbs.116088 40860191 PMC12374839

[125] Li C He M Shi P Yao L Fang X Li X . A novel, rapid, and practical prognostic model for sepsis patients based on dysregulated immune cell lactylation. Front Immunol. (2025) 16:1625311. doi: 10.3389/fimmu.2025.1625311 40612938 PMC12221935

[126] Deng Y Qiu Y Li X Gong T Guo J Liang H . PDK4-driven lactate accumulation facilitates LPCAT2 lactylation to exacerbate sepsis-induced acute lung injury. Cell Death Diff. (2026) 33:557–73. doi: 10.1038/s41418-025-01585-6 41057687 PMC13035903

[127] Chen L Ren R Zhang S Zhu J Li X Zhang X . Pharmacological elevation of lactate alleviates sepsis via histone lactylation-induced IL-10 production. Free Radic Biol Med. (2026) 249:27–42. doi: 10.1016/j.freeradbiomed.2026.03.016 41794152

[128] Xie J Ma R Guo C Li Y Liu X Yu X . HIF-1 A-mediated lactate metabolism confers ferroptosis resistance in M1 macrophages through histone lactylation during acute lung injury. Inflammation. (2026) 49:134. doi: 10.1007/s10753-026-02501-x 41888366 PMC13171684

[129] Yu H Liu M Hou S Wu J Du Q Feng F . Jaceosidin attenuates sepsis-induced myocardial dysfunction by promoting SIRT2-mediated inhibition of histone H3K18 lactylation. Pharm (Basel). (2026) 19:97. doi: 10.3390/ph19010097 41599696 PMC12844931

[130] Liu S Yang T Jiang Q Zhang L Shi X Liu X . Lactate and lactylation in sepsis: a comprehensive review. J Inflammation Res. (2024) 17:4405–17. doi: 10.2147/jir.S459185 39006496 PMC11244620

[131] Liu X Wang H Ni W Dong X Zheng M Chang W . Global lactylome reveals lactylation-dependent mechanisms underlying CXC motif chemokine ligand 12 expression in pulmonary endothelium during acute respiratory distress syndrome. MedComm (2020). (2025) 6:e70344. doi: 10.1002/mco2.70344 40895187 PMC12394890

[132] Xie X Liu T Zhang C Cheng Y Gao Y Xiao W . Targeting the lactylation of ENO1 alleviates endothelial dysfunction in sepsis. Clin Transl Med. (2026) 16:e70597. doi: 10.1002/ctm2.70597 41532698 PMC12801394

[133] Liu J Zhou F Tang Y Li L Li L . Progress in lactate metabolism and its regulation via small molecule drugs. Molecules. (2024) 29:5656. doi: 10.3390/molecules29235656 39683818 PMC11643809

[134] Fang Y Dou A Zhang Y Zhang Y Gao Y Xie K . Research on sepsis and metabolic reprogramming from 1998 to 2025: a bibliometric and visualized analysis. Shock. (2025). doi: 10.1097/shk.0000000000002714 40986928 PMC13384386

[135] Yang K Fan M Wang X Xu J Wang Y Tu F . Lactate promotes macrophage HMGB1 lactylation, acetylation, and exosomal release in polymicrobial sepsis. Cell Death Diff. (2022) 29:133–46. doi: 10.1038/s41418-021-00841-9 34363018 PMC8738735

[136] Li Z Zhang W Zhang G . PROTAC-mediated degradation of Class I HDACs by JPS016 alleviates septic cardiomyopathy via mitophagy-driven exopher formation and mitochondrial quality control. Int J Biochem Cell Biol. (2026) 192:106910. doi: 10.1016/j.biocel.2026.106910 41687730

[137] Sun Z Song Y Li J Li Y Yu Y Wang X . Potential biomarker for diagnosis and therapy of sepsis: lactylation. Immun Inflammation Dis. (2023) 11:e1042. doi: 10.1002/iid3.1042 37904710 PMC10571012

